# Phylogeny and Divergence Times of Gymnosperms Inferred from Single-Copy Nuclear Genes

**DOI:** 10.1371/journal.pone.0107679

**Published:** 2014-09-15

**Authors:** Ying Lu, Jin-Hua Ran, Dong-Mei Guo, Zu-Yu Yang, Xiao-Quan Wang

**Affiliations:** State Key Laboratory of Systematic and Evolutionary Botany, Institute of Botany, Chinese Academy of Sciences, Beijing, China; Royal Botanic Gardens, Kew, United Kingdom

## Abstract

Phylogenetic reconstruction is fundamental to study evolutionary biology and historical biogeography. However, there was not a molecular phylogeny of gymnosperms represented by extensive sampling at the genus level, and most published phylogenies of this group were constructed based on cytoplasmic DNA markers and/or the multi-copy nuclear ribosomal DNA. In this study, we use *LFY* and *NLY*, two single-copy nuclear genes that originated from an ancient gene duplication in the ancestor of seed plants, to reconstruct the phylogeny and estimate divergence times of gymnosperms based on a complete sampling of extant genera. The results indicate that the combined *LFY* and *NLY* coding sequences can resolve interfamilial relationships of gymnosperms and intergeneric relationships of most families. Moreover, the addition of intron sequences can improve the resolution in Podocarpaceae but not in cycads, although divergence times of the cycad genera are similar to or longer than those of the Podocarpaceae genera. Our study strongly supports cycads as the basal-most lineage of gymnosperms rather than sister to Ginkgoaceae, and a sister relationship between Podocarpaceae and Araucariaceae and between Cephalotaxaceae-Taxaceae and Cupressaceae. In addition, intergeneric relationships of some families that were controversial, and the relationships between Taxaceae and Cephalotaxaceae and between conifers and Gnetales are discussed based on the nuclear gene evidence. The molecular dating analysis suggests that drastic extinctions occurred in the early evolution of gymnosperms, and extant coniferous genera in the Northern Hemisphere are older than those in the Southern Hemisphere on average. This study provides an evolutionary framework for future studies on gymnosperms.

## Introduction

A solid organismal phylogeny is fundamental to study evolutionary biology and historical biogeography. In recent years, the angiosperm phylogeny group (APG) III system has provided an evolutionary framework for studying angiosperms [1]. However, phylogenetic relationships of the main lineages of gymnosperms, either classified into four subclasses (Cycadidae, Ginkgoidae, Gnetidae and Pinidae) by Christenhusz *et al.* [2] or into the widely accepted five clades (cycads, ginkgos, cupressophytes, Pinaceae and gnetophytes), are still in hot debate. Gymnosperms, which have been resolved as the sister group of angiosperms by increasing evidence from morphological, molecular phylogenetic and evolutionary developmental studies [3]–[13], bear important information of seed-plant evolution, and represent an important link in the evolution of many gene families and biological pathways. Therefore, a better understanding of evolutionary relationships within gymnosperms can also help us to interpret the evolution of seed plants, and even molecular evolution in land plants.

Gymnosperms have a rich fossil record that is very useful for phylogenetic reconstruction, but this group suffered a dramatic extinction in the Cenozoic [14] and currently comprises 12 families, 83 genera, and only a little more than 1,000 species [2], which makes it difficult to resolve some interfamilial and intergeneric relationships (see review by Wang and Ran [13]). The early molecular phylogenetic studies of gymnosperms only sampled a small part of the recognized genera [4], [5], [15]–[17], and in particular most published molecular phylogenies were constructed based on uniparentally inherited cytoplasmic DNA markers and/or the multi-copy nuclear ribosomal DNA [4], [5], [14]–[16]. Despite that 53 genera representing all extant main lineages of gymnosperms were studied in Ran *et al.* [16], the main focus of the study was the fast evolution of the mitochondrial gene *rps*3 in Conifer II (cupressophytes) and the underlying mechanisms. Some other studies of gymnosperms mainly focused on individual families or clades, such as conifers [17], [18], Cupressaceae [9]–[22], Pinaceae [23] and cycads [24], . Although great progress has been made on understanding the phylogeny of gymnosperms in recent years, more interesting phylogenetic hypotheses have been proposed and hotly debated (see review by Wang and Ran [13]), like the phylogenetic position of Gnetales and the relationship between cycads and ginkgos [11], [12], [26], . Till now, there is not a molecular phylogeny of gymnosperms that is reconstructed based on a complete sampling of all extant genera, although this ancient and widespread plant group has huge ecological and economic value. Also, it would be interesting to know whether the phylogenetic relationships of gymnosperms inferred from cytoplasmic DNA are supported by evidence from the nuclear genome, given the complex inheritance patterns of organellar genes in this group [28]. Moreover, phylogenetic relationships within some lineages, such as Pinaceae [23], [29], Podocarpaceae [30] and Zamiaceae [25], [31], [32], need to be further resolved.

Due to the fast development of genome sequencing technologies, phylogenomic analyses have been increasingly used in reconstructing the tree of life, and the efficiency of using multiple single- or low-copy nuclear genes for phylogenetic analysis has been widely recognized [33]. However, this is still difficult for gymnosperms with large and complex nuclear genomes characterized by long introns and numerous gene-like fragments [34]. For example, based on ESTs, Lee *et al.* [27] analyzed millions of amino acid sites from 150 species across land plants, and placed Gnetales as sister to the rest of the gymnosperms, but their dataset suffered greatly from missing data and poor alignment (our unpublished analysis). Nevertheless, Yang *et al.* [22] successfully used two sister nuclear genes *LEAFY* (*LFY*) and *NEEDLY* (*NLY*), which originated from an ancient gene duplication in the common ancestor of seed plants and encode transcription factors regulating the development of reproductive structures in gymnosperms [35], [36], to reconstruct the phylogeny of Cupressaceae comprising all its 32 genera. They also confirmed that both *LFY* and *NLY* exist as single copy in gymnosperms, even in the polyploid species, and are excellent markers for studying the phylogeny and evolution of gymnosperms [22].

In this study, on the basis of Yang *et al.* [22], we use *LFY* and *NLY* gene sequences to reconstruct the phylogeny of gymnosperms based on a complete sampling of extant genera, in effort to provide an evolutionary framework for future studies on this important group. In addition, some controversial interfamilial and intergeneric relationships are resolved and discussed. Moreover, benefiting from the rich fossil record, we estimate the divergence times of different lineages, which would further help us understand the diversification history of gymnosperms.

## Materials and Methods

### Ethics statement

No specific permits were required for the sampling.

### Taxon sampling

Ninety species representing all recognized genera of extant gymnosperms were sampled. Most genera were represented by one species each, since the coding sequences of *LFY* and *NLY* used to reconstruct the phylogeny of gymnosperms are very conserved among congeneric species. If using introns of the two genes, the sequences are unalignable between the main clades of gymnosperms [22], and most congeneric species do not form monophyletic groups, respectively, due to the wide interspecific sharing of alleles as reported in *Pinus* [37]. Therefore, the addition of more congeneric species can not significantly improve the resolution of intergeneric relationships of gymnosperms when using single-copy nuclear genes like *LFY* and *NLY*. Nevertheless, we sampled two species of *Pinus* to represent its two subgenera with an ancient divergence, and more species from the *Juniperus*-*Cupressus*- *Callitropsis-Xanthocyparis-Hesperocyparis* clade, in which the generic division is controversial [22]. The origins of materials, including the data downloaded from NCBI, are shown in Table S1.

### DNA and RNA extraction, PCR and RT-PCR amplification, cloning and sequencing

Total DNA was extracted from silica-gel dried leaves using either the modified CTAB method [38] or the DNAsecure Plant Kit (Tiangen, Beijing, China). Young leaves and reproductive organs of *Ephedra equisetina* were collected for total RNA extraction, which followed the modified Trizol method (Tiangen). The first-strand cDNA was produced using the 5′ RACE system (Invitrogen) and the 3′ RACE kit (Tiangen). Polymerase chain reaction (PCR) was conducted in a Veriti 96-Well Thermal Cycler (Applied Biosystems, Foster City, CA, USA) or an Eppendorf Mastercycler (Eppendorf Scintific, Westbury, NY, USA), in a volume of 25 µl containing 50–200 ng of DNA or cDNA template, 6.25 pmol of each primer, 0.2 mM of each dNTP, 2 mM MgCl_2_, and 0.75 U of ExTaq DNA polymerase (Takara Biotechnology, CO., Ltd. Dalian, China). PCR cycles were as follows: one cycle of 4 min at 94°C, four cycles of 1 min at 94°C, 30 s at 55–58°C, and 1.5–6.0 min at 72°C, followed by 32 cycles of 30 s at 94°C, 30 s at 53–55°C and 1.5–6.0 min at 72°C, with a final extension step for 10 min at 72°C.

After separation by 1.5% agarose gel electrophoresis, the PCR products were purified using the TIANgel Midi Purification Kit (Tiangen) and identified by direct sequencing with the PCR primers. Then, the correct PCR products were cloned with the pGEM-T Easy Vector System II (Promega, Madison, USA). Ten clones with the correct insertion, confirmed by *Eco*R I digestion, were picked for each species and screened for variation by sequencing with T7 primer. All distinct clones were further sequenced using SP6 and internal primers. Sequencing was performed using the BigDye Terminator v3.1 Cycle Sequencing Kit., and the sequencing products were separated on a 96-capillary 3730XL DNA analyzer (Applied Biosystems). All newly sequenced *LFY* and *NLY* genes, totaling 104 sequences, are deposited in NCBI under GenBank accession numbers KF377856-KF377901, KF377904-KF377918 and KF377921-KF377963 (Table S1). The primers used for amplifying and sequencing the *LFY* and *NLY* genes are shown in Table S2.

### DNA sequence analysis

Sequence alignments were generated with CLUSTAL X [39] and manually refined. The variable sites and variability of conspecific clones were calculated using MEGA5 [40] and BioEdit v7.2.0 [41], respectively. Introns of the two nuclear genes could not be reliably aligned among distantly related gymnospermous families, and thus were excluded when constructing the entire phylogeny of gymnosperms. However, some intron regions are relatively conserved and alignable within cycads and Podocarpaceae, respectively, and thus were included in the alignments to infer the intergeneric relationships of these groups. The aligned sequences were further trimmed using the Gblocks server (http://molevol.cmima.csic.es/castresana/Gblocks_server.html).

We used the software DAMBE [42] to test substitution saturation for the two datasets *LFY* and *NLY*, and the results showed that none of them was substitutionally saturated. To determine whether the two gene datasets can be combined, we checked variation of clones in each species, and found that many species did not show clone polymorphism of *LFY* and *NLY* and no more than two distinct clones occurred in the same individual. In particular, the conspecific clones showed a high sequence similarity of over 95%. Then, we tried to conduct separate phylogenetic analyses for *LFY* and *NLY* that included all distinct clones, and the results showed that conspecific clones grouped together except two *LFY* clones from the tetraploid species *Fitzroya cupressoides* that were discussed in Yang *et al.* [22]. Therefore, we randomly selected one clone from each species for the further analyses. The incongruence length difference test (ILD) [43], implemented in PAUP^*^ 4.0b10 [44], CONCATERPILLAR (a hierarchical likelihood ratio test) [45], and CADM (a test of congruence among distance matrices) [46] were performed to assess congruence between different datasets. According to the three tests, no significant incongruence existed between *LFY* and *NLY* (Table 1), so we combined the two genes for phylogenetic analysis.

**Table 1:** Results of the ILD, CADM, and CONCATERPILLAR tests.

Datasets	ILD	CADM		CONCATERPILLAR	
	p-value	ω	Prob.perm	Raw p-value	Weibull-smoothed p-value
Gymnosperms (1)					
CDS	0.256	0.977	0.001	0.25	0.248
CDS (1^st^+2^nd^)	0.417	0.965	0.001	0.13	0.145
Gymnosperms (2)					
CDS	0.292	0.970	0.001	0.53	0.575
CDS (1^st^+2^nd^)	0.356	0.953	0.001	0.24	0.265
Taxaceae+Cephalotaxaceae					
CDS	0.066	0.912	0.002	0.28	0.255
Cycads					
CDS	0.259	0.960	0.001	0.52	0.413
CDS+Intron	0.478	0.939	0.001	0.80	0.822
Podocarpaceae					
CDS	0.887	0.898	0.001	0.28	0.298
CDS+Intron	0.005	0.870	0.001	0.01	0.019

Gymnosperms (1): *Angiopteris* as outgroup; Gymnosperms (2): Cycads as functional outgroups; CDS: coding sequence; 1^st^+2^nd^: the first and second codon positions.

### Phylogenetic analysis

Initially, we used the *LFY* + *NLY* coding sequences (CDS) and the 1^st^+2^nd^ codon positions, respectively, to reconstruct the phylogeny of all sampled gymnosperms. The fern *Angiopteris lygodiifolia* was used as outgroup for two reasons. First, as mentioned in the introduction, the two nuclear genes of gymnosperms originated from a duplication event in the common ancestor of seed plants, and the *NLY* gene was lost in angiosperms. Thus, the *LFY* gene of ferns may represent an ancestral state of the two genes. Second, the *LFY* gene sequence cannot be reliably aligned between gymnosperms and angiosperms, although a sister relationship between the two groups is supported by most recent studies (see review by Wang and Ran [13]). The results showed that the phylogenetic trees generated from different methods all supported cycads as a monophyletic group and the basal-most clade of gymnosperms (Fig. S1). To avoid long-branch attraction (LBA) artifacts, the phylogeny of gymnosperms was further reconstructed using cycads as functional outgroups. In addition, to better resolve the intergeneric relationships within cycads and Podocarpaceae that were controversial, we conducted separate phylogenetic analyses for the two lineages with combined *LFY* + *NLY* sequences, and compared gene trees generated from CDS and CDS+intron, respectively. The sister groups were chosen as outgroups, including *Ginkgo biloba* for cycads [26], as well as *Araucaria heterophylla* and *Agathis robusa* for Podocarpaceae [22]. When introns were included, the sequences could not be aligned among different gymnospermous families, and therefore the generated trees were not rooted or rooted with a functional outgroup, such as *Cycas* for cycads. The details of all datasets used for phylogenetic analyses are shown in Table 2. The trees and alignments are deposited in TreeBase (number S16207).

**Table 2:** Datasets used for phylogenetic analyses and model settings as determined in jModeltest 2.0 and MrModeltest 2.3 using Akaike Information Criterion (AIC).

Dataset		Best model for ML	Best model for BI	Sequence information		
				Length	No. of variable sites	No. of informative sites
**Gymnosperms (1)**						
*LFY*	CDS	TIM3+I+G	GTR+I+G	999	615	518
	CDS (1^st^+2^nd^)	TIM3+G	GTR+I+G	666	299	210
*NLY*	CDS	GTR+I+G	GTR+I+G	948	571	497
	CDS (1^st^+2^nd^)	GTR+G	GTR+I+G	632	274	208
*LFY*+*NLY*	CDS	TIM3+I+G	GTR+I+G	1947	1186	1015
	CDS (1^st^+2^nd^)	GTR+G	GTR+I+G	1298	573	418
**Gymnosperms (2)**						
*LFY*	CDS	TIM3+I+G	GTR+I+G	990	566	457
	CDS (1^st^+2^nd^)	TIM3+G	GTR+I+G	660	251	153
*NLY*	CDS	GTR+I+G	GTR+I+G	945	530	447
	CDS (1^st^+2^nd^)	TIM3+I+G	GTR+I+G	630	235	163
*LFY*+*NLY*	CDS	TIM3+I+G	GTR+I+G	1935	1096	904
	CDS (1^st^+2^nd^)	GTR+G	GTR+I+G	1290	486	316
**Cycads**						
*LFY*	CDS+Intron	GTR+I	GTR+I	2014	738	229
	CDS	TIM3+G	GTR+G	1121	311	110
*NLY*	CDS+Intron	TIM3+G	GTR+G	1140	400	152
	CDS	TIM3+G	GTR+G	992	339	137
*LFY*+*NLY*	CDS+Intron	GTR+G	GTR+G	3154	1138	381
	CDS	TIM3+G	GTR+I+G	2113	650	247
**Podocarpaceae**						
*LFY*	CDS+Intron	TIM3+I+G	GTR+G	1981	905	429
	CDS	TIM3+G	GTR+I+G	1156	403	239
*NLY*	CDS+Intron	GTR+I+G	GTR+I+G	3134	1613	678
	CDS	TrN+I+G	GTR+I+G	967	295	169
*LFY*+*NLY*	CDS+Intron	GTR+I+G	GTR+I+G	5155	2518	1107
	CDS	TIM3+I+G	GTR+I+G	2123	698	408
**Taxaceae+Cephalotaxaceae**						
*LFY*	CDS	TIM3+G	GTR+I	1104	218	84
*NLY*	CDS	TrN+I	GTR+G	967	162	64
*LFY*+*NLY*	CDS	TrN+I	GTR+G	2071	380	148

Gymnosperms (1): *Angiopteris* as outgroup; Gymnosperms (2): Cycads as functional outgroups; CDS: coding sequence; 1^st^+2^nd^: the first and second codon positions.

Phylogenetic relationships were reconstructed using maximum parsimony (MP), maximum likelihood (ML) and Bayesian inference (BI), respectively. The MP analyses were implemented in PAUP^*^ 4.0b10 [44], using heuristic searches with 1000 random addition sequence replicates, starting trees obtained via stepwise addition, tree-bisection-reconnection (TBR) branch swapping, MulTrees and Collapse options in effect, and a maximum of 2000 trees saved for each replicate. Robustness of the nodes (50% majority-rule consensus) was tested by the bootstrap analysis [47] using 1000 replicates with the same settings as above. The evolutionary models for the ML and BI analyses were optimized in jModeltest 2.0 [48] and MrModeltest 2.3 [49], using Akaike Information Criterion (AIC), respectively. The best models for analyses are shown in Table 2. The ML analyses were carried out in PHYML version 2.4.4 [50] with a BIONJ tree as a starting point, and support values for the nodes were calculated based on 100 bootstrap replicates. The Bayesian inference was performed with MrBayes 3.1.2 [51]. One cold and three heated Markov chain Monte Carlo chains were run for 10,000,000 generations with random initial trees, and every 1000 generations were sampled. The first 20% of the samples were discarded as burn-in and a 50% majority-rule consensus tree was generated based on the trees sampled after generation 2,000,000.

The Shimodaira-Hasegawa test (SH test) [52] and the Kishino-Hasegawa test (KH test) [53], implemented in PAUP^*^ 4.0b10, were used to test alternative phylogenetic hypotheses for the deep lineages with controversial phylogenetic positions. The different positions of three taxa, including Ginkgoaceae (sister to cycads or conifers + Gnetales), Gnetales (sister to conifers, Conifer II, Pinaceae, or other gymnosperms), and Sciadopityaceae (sister to Cupressaceae + Taxaceae + Cephalotaxaceae or Araucariaceae + Podocarpaceae), were compared. Alternative tree topologies were generated in PhyML 2.4.4 [50], and the tree files were run in PAUP to calculate the p-value for each topology.

### Divergence time estimation

Based on the *LFY* + *NLY* coding sequences, the divergence times of gymnosperms were estimated using the Markov chain Monte Carlo (MCMC) method, which was implemented in BEAST v1.7.5 [54], under an uncorrelated lognormal-relaxed clock model of rate variation among lineages. The topology was constrained to reflect the ML tree, and a GTR+I+G substitution model was used. Mean substitution rates were allowed to vary. Sauquet *et al.* [55] suggested that more age constraints could lead to improved time estimates, but risky age constraints might strongly influence estimated ages. Hence, we incorporated 11 fossil constraints that were widely recognized and used in previous molecular dating of gymnosperms or seed plants [18], [22], [56], and nearly each main lineage of gymnosperms was calibrated by at least one fossil record (For details, see Table S3).

For the most recent common ancestor (MRCA) of gymnosperms (A), a minimum age of 306.2 Ma was set based on *Cordaixylon iowensis*, the oldest cordaitean coniferophyte found in the Laddsdale Coals (Cherokee Group, Desmoinesian Series; 307.2±1.0 Ma) near What Cheer of Iowa, and a maximum age of 366.8 Ma was set based on the well-documented first appearance of seeds (in the form of preovules) in the Upper Fammenian (Upper Devonian) VCo Spore Biozone [57]–[59]. In cycads, the stem age of *Lepidozamia* (B) was constrained to a minimum age of 33.9 Ma based on the fossil of *Lepidozamia* leaves from the Eocene of Australia, which possesses cuticular characters that are unique to *Lepidozamia* [60]. In the family Pinaceae, the stem age of *Picea* (C) was constrained by *Picea burtonii* from the Apple Bay locality, Vancouver Island, British Columbia, dated to the Valanginian Stage of the Early Cretaceous (≥133 Ma). This seed cone fossil shares multiple morphological and anatomical characteristics with extant *Picea*, especially in the distribution and branching pattern of resin canals in the ovule scale [61]. For Gnetales, its crown node (D) was calibrated based on *Eoantha zherikhinii* (≥125 Ma), which is a reproductive organ with whorls of scales and is considered closely related to *Gnetum* and *Welwitschia* [62], [63]. In Conifer II, we set a minimum age of 172 Ma for the Araucariaceae-Podocarpaceae split (E) based on the first appearance of *Araucarites phillipsii*-*Brachyphyllum mammilare* from the Aalenian (172–176 Ma) [64], and 28 Ma for the *Podocarpus*-*Retrophyllum* split (F) based on *Retrophyllum australe* from the West Dale Flora of southwestern Australia (dated to 28–48 Ma) [65]. The two calibrations were also used in Leslie *et al.* [18]. *Araucarites phillipsii*, with seed cones similar to those in Araucariaceae, was considered as the first unambiguous evidence for the stem or crown of the plant family, and *Brachyphyllum mammilare* was found to have pollen cones that produced relatively large, non-saccate pollen comparable to modern *Araucaria* and foliages that contained oval sclereids similar to those in extant *Araucaria cunninghamii*. In addition, *Retrophyllum australe* had distinctive heterofacially flattened foliage similar to *Nageia* and *Afrocarpus*. For the split of Taxaceae-Cupressaceae (G), a minimum age of 197 Ma was set based on *Palaeotaxus rediviva* from the Skromberga Colliery in Scania, Sweden (dated to 197–201 Ma) [66], which showed an axillary short shoot that terminated in a single ovule and bore helically arranged sterile scales on seed cones identical to extant *Austrotaxus* and *Taxus*. The remaining fossil constraints were used to set a minimum age for four nodes in Cupressaceae *s.l.*, as in Yang *et al.* [22], including the MRCAs of *Sequoia-Metasequoia-Sequoiadendron* (H, 140 Ma, *Sequoia* in early Cretaceous) [67], [68], *Glyptostrobus-Taxodium* (I, 99 Ma, *Glyptostrobus* in Cretaceous) [69], [70], *Diselma-Fitzroya-Widdringtonia* (J, 95 Ma, *Widdringtonia* in Cretaceous) [71], and *Juniperus-Cupressus-Hesperocyparis* (K, 33.9 Ma, *Juniperus* in the Eocene/Oligocene boundary) [72].

Since the age estimates by BEAST are usually older than those by PL (penalized likelihood) and the ages estimated with lognormal priors are slightly younger than those estimated with either uniform or exponential priors, Sauquet *et al.* [55] suggested that using lognormal priors can decrease the uncertainty in age estimates. Therefore, in this study, all fossil constraints were given lognormal prior distributions in the BEAST estimate. For the root constraint, we used a stdev of 0.5, a prior mean of 3.6, and an offset of 290.7 Ma. For a better comparability of our results with previous divergence time estimates of gymnosperms, the other constraints were set following Leslie *et al.* [18] and Yang *et al.* [22]. The minimum age was set by the age of fossil, with a 95% confidence interval of the probability distribution extending 20 or 40 million years earlier than this minimum age, since the test by Leslie *et al.* [18] found that the fossil calibrations associated with the two prior age distributions led to very similar divergence time estimates. We ran four independent MCMC runs of 100 million generations, sampling every 2,500 generations. Tracer v1.5 was used to check convergence of the chains to the stationary distribution, ensuring the Effective Sample Size (ESS) >200. The first 20% of the generations were discarded as burn-in and trees were summarized with TreeAnnotator. The final tree and divergence times were visualized using FigTree v1.4.0.

## Results

### Sequence characterization

In this study, we cloned and sequenced the *LFY* and *NLY* genes from 41 genera of 7 families (Table S1). These new data combined with the sequences downloaded from GenBank (mostly reported in Yang *et al.* [22]) completely represented all extant genera of gymnosperms. In *Parasitaxus usta*, the only parasitic conifer, we only got a pseudogene of *NLY*, in which several indels in the second exon led to an ORF shift. It is interesting that, by RT-PCR, we obtained cDNA sequences of both *LFY* and *NLY* genes from *Ephedra equisetina* and the *LFY* gene had two clone types that differed by a 9-bp deletion.

Both *LFY* and *NLY* sequences amplified from genomic DNA comprised three exons and two introns, and almost covered the full length of the two genes. The exon length was conserved, totally about 1000 bp, but the intron length varied greatly among different groups. A long repeat occurred in the first intron of the *NLY* gene of four Taxaceae genera (*Pseudotaxus, Austrotaxus, Amentotaxus and Torreya*), making it difficult to sequence the full length of the gene. Also, the first *NLY* intron of *Cathaya* and *Pseudotsuga*, two genera of the pine family, was difficult to sequence due to long length or complex structures. The detailed information of the sequence alignments for phylogenetic analyses, including sequence lengths and numbers of variable and parsimony-informative sites, is shown in Table 2.

### Phylogenetic analysis

**Figure 1:**
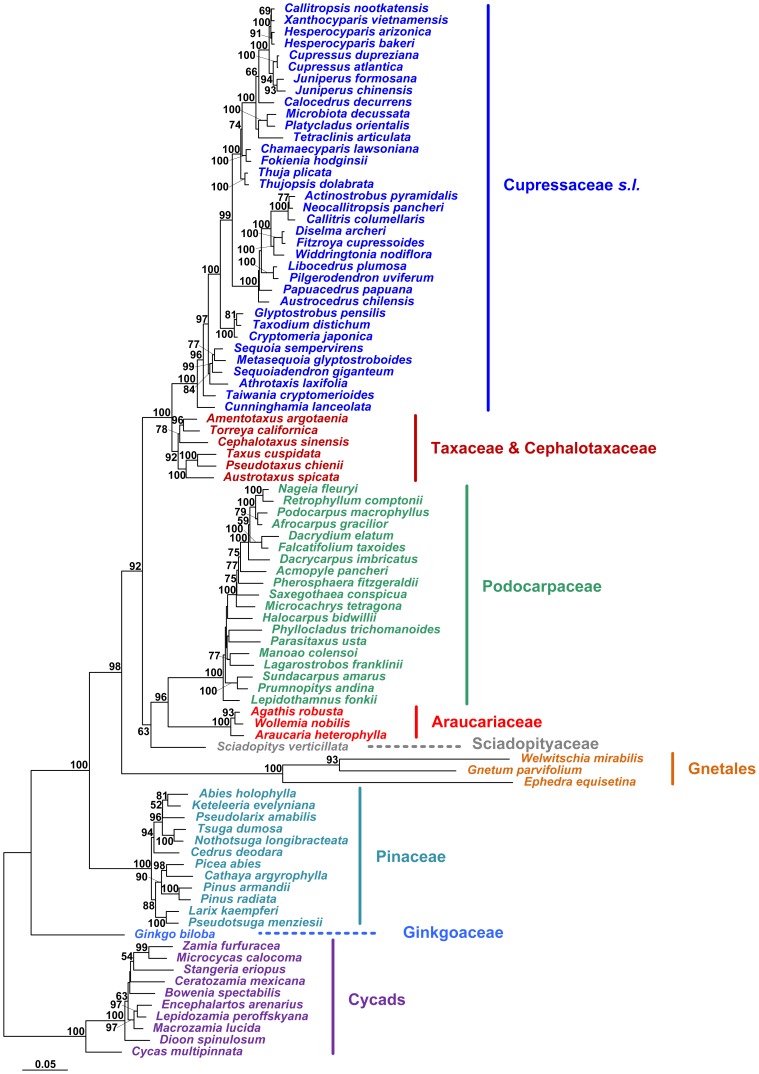
The ML tree of gymnosperms constructed from combined *LFY* and *NLY* CDS sequences. Numbers associated with branches are bootstrap percentages higher than 50%. The cycads were used as functional outgroups.

Since the MP analysis is more easily affected by long branch attraction (LBA) than the ML and BI analyses [73]–[75], we did not show the MP trees in this study. As mentioned earlier, when *Angiopteris lygodiifolia* was used as outgroup, all phylogenetic trees generated supported cycads as a monophyletic and basal-most group of gymnosperms, followed by *Ginkgo* (see the ML and BI trees in Fig. S1). When cycads were used as functional outgroups, the ML and BI trees generated from combined *LFY* and *NLY* CDS were topologically identical to each other, except for some branches with low statistical support. In the ML tree (Fig. 1), Ginkgoaceae was sister to the remaining gymnosperms excluding cycads, and Pinaceae was sister to a clade that was further divided into two sister subclades, i.e., Gnetales and conifer II (Cupressophytes). The conifer II was split into two lineages. One consisted of Sciadopityaceae, Podocarpaceae and Araucariaceae, and Sciadopityaceae was weakly supported to be sister to Podocarpaceae-Araucariaceae. Within the other lineage, Cephalotaxaceae was embedded in Taxaceae, and the two families formed a monophyletic group sister to Cupressaceae. In addition, this nuclear gene tree provided a relatively good resolution for intergeneric relationships in some families such as Pinaceae and Cupressaceae.

**Figure 2:**
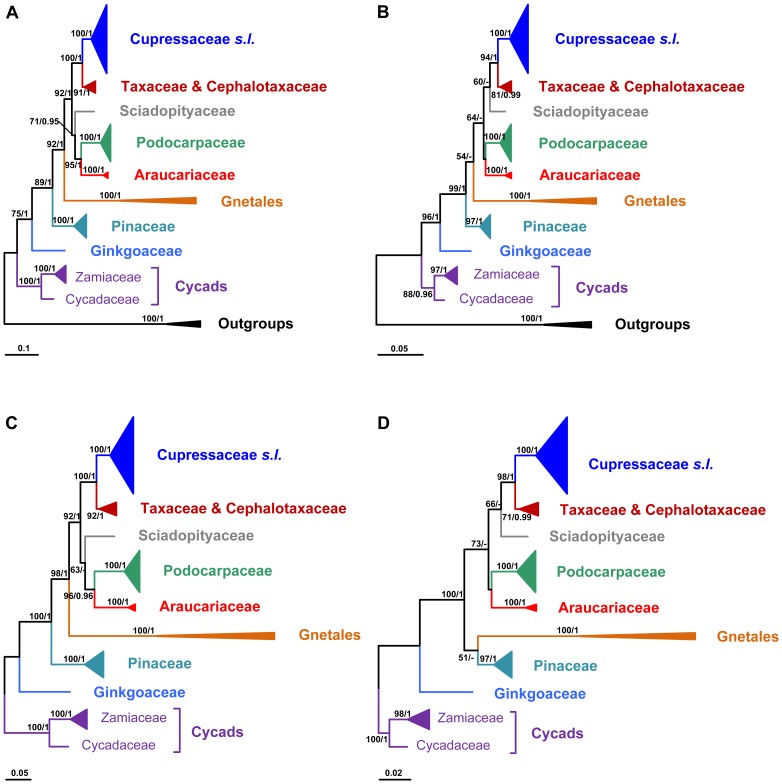
Comparison of ML trees of gymnosperms constructed using *LFY* + *NLY* sequences. A and C, All three codon positions were used; B and D, 1^st^ and 2^nd^ codon positions were used. A and B, *Angiopteris lygodiifolia* was used as outgroup; C and D, The cycads were used as functional outgroups. Numbers associated with branches are bootstrap percentages of ML higher than 50% and Bayesian posterior probabilities greater than 0.90, respectively.

Although the trees generated with different rooting or from different codon positions (all CDS vs. 1^st^+2^nd^ codons) were similar in topology, they differed in the positions of Gnetales and Sciadopityaceae (Fig. 2). For instance, in the phylogenetic tree generated from the first and second codon positions and rooted with cycads (Fig. 2D), Gnetales was weakly supported to be sister to Pinaceae, and Sciadopityaceae sister to a well-supported clade containing Cupressaceae and Taxaceae-Cephalotaxaceae. Moreover, many intra-familial relationships were poorly resolved (tree not shown), perhaps due to the declined phylogenetic signals caused by the removal of the third codon positions.

**Figure 3:**
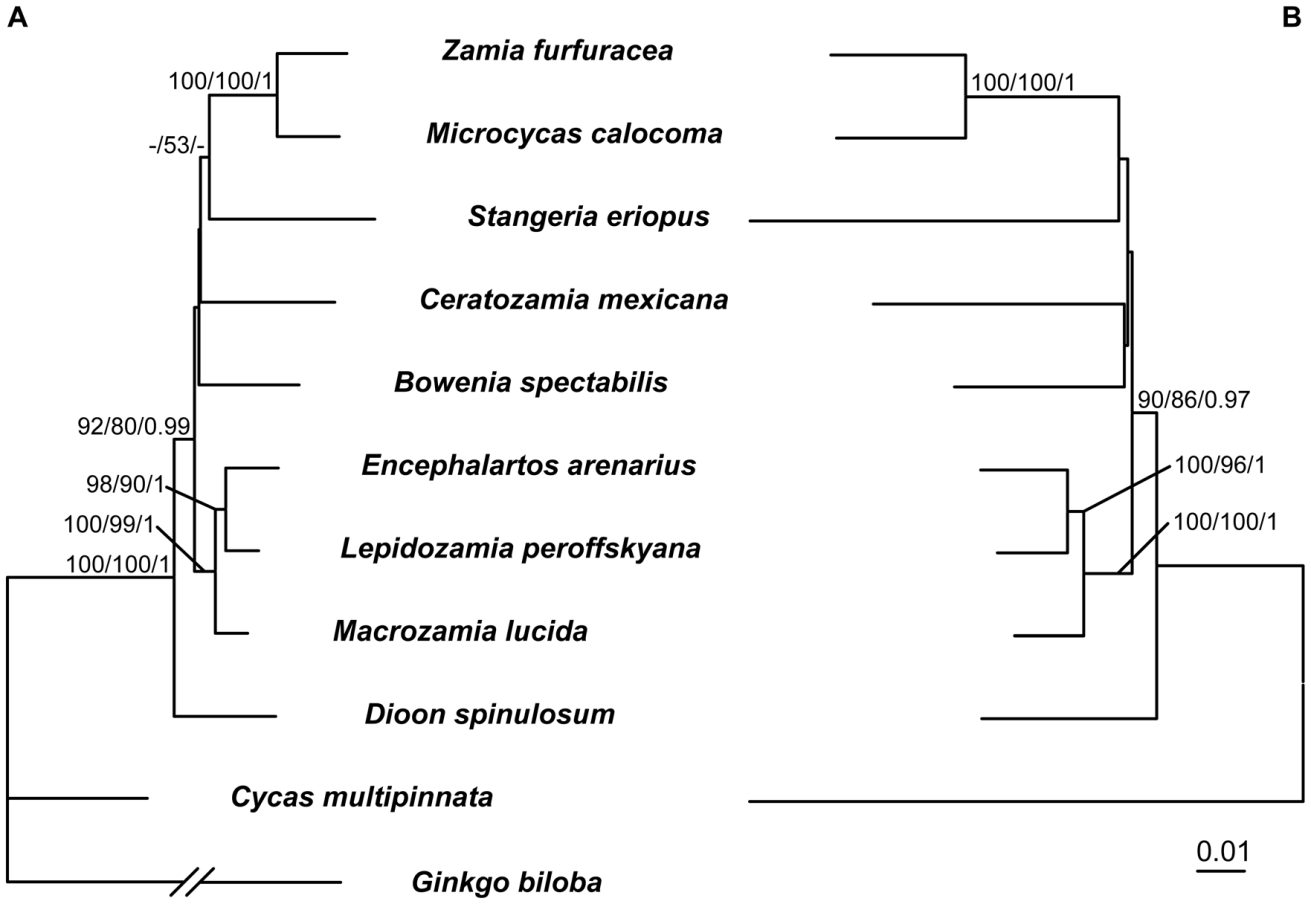
The ML trees of cycads inferred from sequence analysis of combined *LFY* and *NLY* sequences. A, CDS; B, CDS+Intron. Numbers associated with branches are bootstrap percentages of ML and MP higher than 50% and Bayesian posterior probabilities greater than 0.90, respectively. *Ginkgo biloba* was used as outgroup in Fig. 3A.

**Figure 4:**
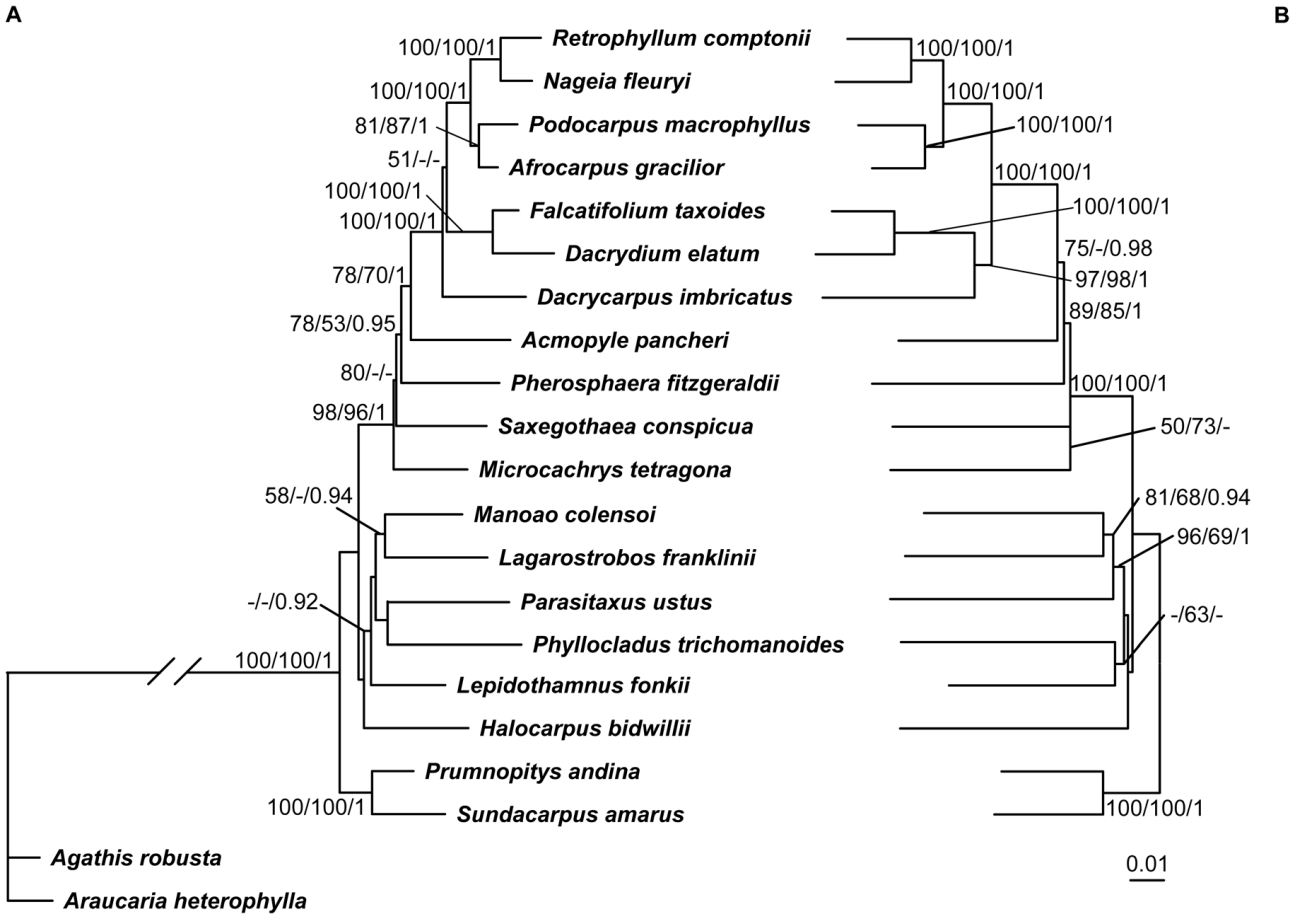
ML trees of Podocarpaceae constructed from sequence analysis of combined *LFY* and *NLY* sequences. A, CDS; B, CDS+Intron. Numbers associated with branches are bootstrap percentages of ML and MP higher than 50% and Bayesian posterior probabilities greater than 0.90, respectively. *Araucaria heterophylla* and *Agathis robusa* were used as outgroups in Fig. 4A.

Since the phylogenetic positions of some genera of cycads and Podocarpaceae were controversial in previous studies [14], [24], [30]–[32], [76]–[79], here we reconstructed internal relationships of the two groups, respectively. When only the *LFY* and *NLY* CDS was used, the phylogenetic signals were insufficient to resolve some intergeneric relationships (Figs. 3, 4), therefore we added the conserved intron regions of the two genes into analysis. For cycads, the addition of introns neither changed the tree topology nor greatly improved the resolution (Fig. 3), and the generated trees suggested a basal position of *Dioon* in Zamiaceae, a sister relationship between *Zamia* and *Microcycas*, and a close relationship among *Encephalartos*, *Lepidozamia* and *Macrozamia*. However, the resolution of internal relationships of Podocarpaceae was improved by adding intron sequences, with high support values for most nodes (Fig. 4). A large clade was strongly supported and well resolved, containing *Microcachrys*, *Saxegothaea*, *Pherosphaera*, *Acmopyle*, *Dacrycarpus*, *Dacrydium*, *Falcatifolium*, *Afrocarpus*, *Podocarpus*, *Nageia* and *Retrophyllum*. Within the clade, there existed two monophyletic sister groups. One was the ‘dacrydioid’ group comprising *Dacrycarpus, Dacrydium* and *Falcatifolium*, and the other was the ‘podocarpoid’ group including the *Retrophyllum-Nageia* subclade and the *Afrocarpus-Podocarpus* subclade. In addition, a close relationship among *Manoao*, *Lagarostrobos* and *Parasitaxus* was revealed (Fig. 4).

The results of the SH and KH tests are shown in Table S4. The trees placing Ginkgoaceae with conifers + Gnetales were better than the trees placing the family sister to cycads, but the trees placing Sciadopityaceae with Podocarpaceae + Araucariaceae were not significantly different from the trees placing Sciadopityaceae sister to Cupressaceae + Taxaceae + Cephalotaxaceae. The sister relationship between Gnetales and the other gymnosperms was rejected by both SH and KH tests for the CDS dataset, and by the KH test for the dataset of the 1^st^+2^nd^ codon positions. In addition, the topology placing Gnetales sister to conifers was rejected by the KH test for the CDS dataset. There was not significant difference in ln score between the other two topologies (Gnetales sister to Conifer II or Pinaceae).

### Divergence time estimation

**Figure 5:**
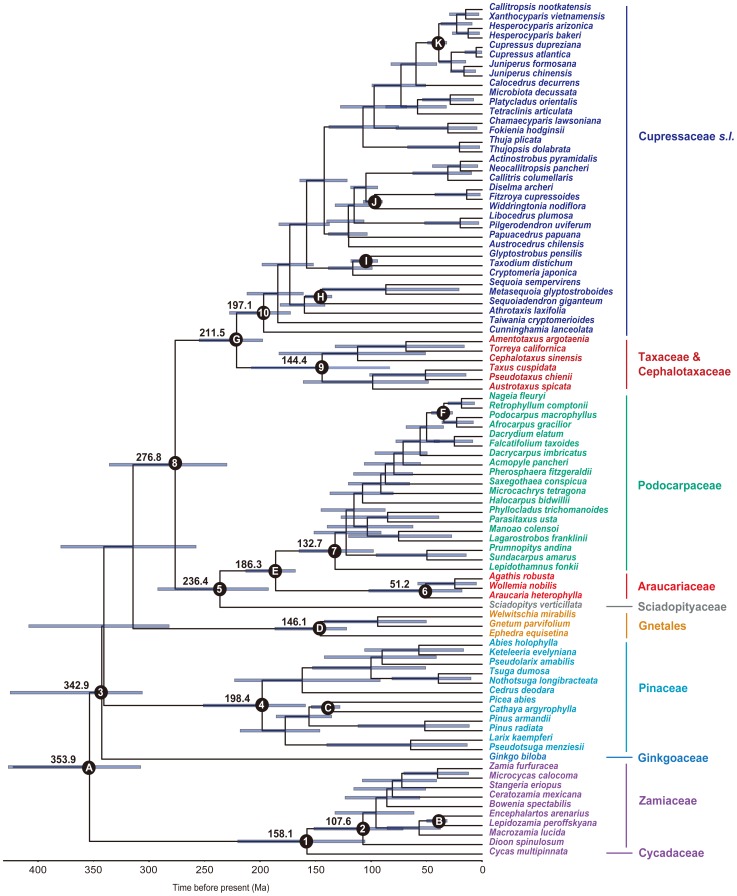
Divergence times of gymnosperms estimated from combined *LFY* and *NLY* CDS sequences using BEAST. A time scale is shown at the bottom. A–K indicate fossil calibration points. 1–10, A, D, E and G indicate some nodes of interest. Median ages of nodes are shown, with horizontal bars indicating the 95% highest posterior density intervals.

The divergence time estimation based on combined *LFY* and *NLY* CDS suggested a Triassic-Jurassic origin of the crown group for most families (Fig. 5). The mean ages and 95% HPDs are shown in Table S5. The most recent common ancestor (MRCA) of cycads was dated to the Middle Jurassic (158.1 Ma), and that of Pinaceae to the Lower Triassic (198.4 Ma). The divergence time between Cupressaceae and Taxaceae *s.l.* was close to that between Podocarpaceae and Araucariaceae, i.e., in the Late Triassic to the Early Jurassic. Most gymnosperm genera originated in the Cretaceous to the Cenozoic (Fig. 5).

## Discussion

### Evolution and phylogenetic utility of the *LFY* and *NLY* genes in gymnosperms

Our study indicates that both *LFY* and *NLY* genes occur in all extant genera of gymnosperms except that *NLY* has not been found in *Gnetum* (Table S1). Frohlich and Parker [80] found that the *LFY*-*NLY* gene pair originated from a duplication event in the common ancestor of seed plants, and then both paralogous genes were remained in gymnosperms while *NLY* was lost in angiosperms. Using this information of gene evolution as strong evidence, they also proposed the mostly male theory of flower origin given the important role of *LFY* in flower development, although this theory is not supported by the study of Vazquez-Lobo *et al.* [35]. The study of Frohlich and Parker [80] only sampled a few species from gymnosperms, and supposed the existence of *NLY* in *Gnetum*. Frohlich [81] further mentioned the occurrence of both *LFY* and *NLY* in *Ephedra* (his unpublished observations), a close relative of *Gnetum*. Our present study has covered all extant gymnospermous genera, and the results suggest that each studied species harbors both *LFY* and *NLY* genes, except that *NLY* is still not found in *Gnetum*. Therefore, our study further supports that the *LFY*-*NLY* gene pair originated from an ancient gene duplication, at least before the divergence of gymnosperms. In addition, we have successfully obtained the *LFY* and *NLY* genes of *Ephedra* by RT-PCR and RACE. Moreover, the selection test suggests that both *LFY* and *NLY* genes have experienced strong purifying selection in gymnosperms (our unpublished data), implying their conserved functions.

Currently, functions of *LFY* and *NLY* in gymnosperms are still not very clear [35], [82]–[87], but it is clear that both of them exist as single-copy genes suitable for phylogenetic reconstruction in gymnosperms based on the present study and Yang *et al.* [22]. No more than two distinct clones were found in the same individual. Although the *NLY* sequence obtained from the parasitic *Parasitaxus usta* represents a pseudogene, its exon region shares a high similarity (over 90%) with that of other Podocarpaceae species, and thus still could be used in phylogenetic analysis. Actually, the *LFY* gene has been successfully utilized in phylogenetic and biogeographic studies of several gymnosperm groups, including *Gnetum* [88], *Thuja* [89], and *Pseudotsuga* [90]. In particular, the intergeneric relationships of Cupressaceae *s.l.* have been well resolved by the *LFY* and *NLY* genes [22].

### Interfamilial relationships of gymnosperms

Our study provides the first molecular phylogeny of gymnosperms covering all extant families and genera, and the phylogeny is based on two single-copy nuclear genes *LFY* and *NLY* (Fig. 1). This nuclear gene phylogeny is topologically largely consistent with most previous phylogenies of gymnosperms constructed based on cytoplasmic and/or nuclear ribosomal DNA [4], [14], [16]. That is, the cycads diverged first, followed by Ginkgoaceae, and then conifers plus Gnetales. Within conifers, Podocarpaceae is sister to Araucariaceae, and Cephalotaxaceae-Taxaceae sister to Cupressaceae (Fig. 2). However, we did not found a sister relationship between cycads and Ginkgoaceae as suggested by chloroplast phylogenomic analyses [12], [26] as well as genome-scale nuclear and plastid data [11].

**Figure 6:**
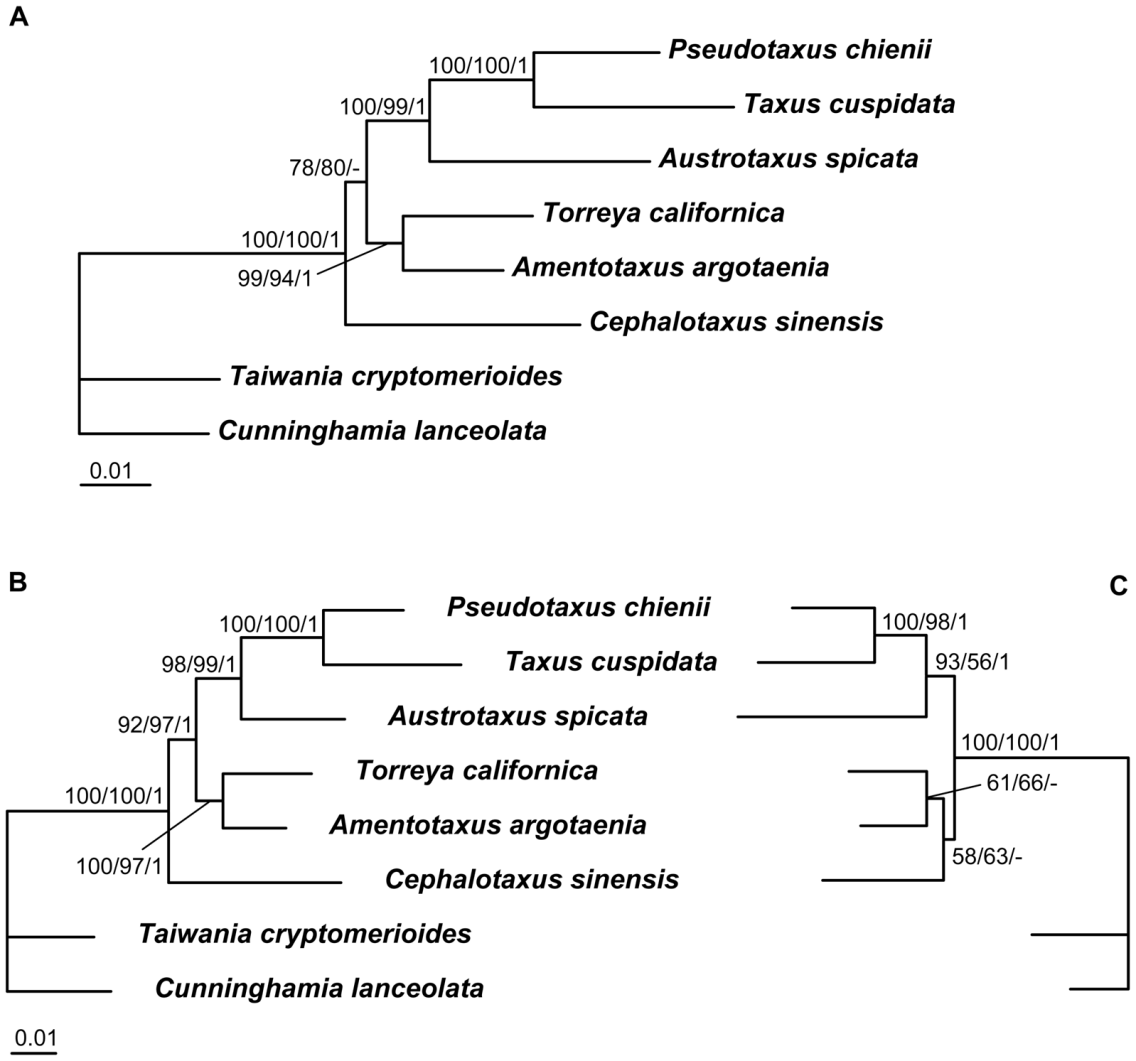
The ML trees of Taxaceae+Cephalotaxaceae constructed from CDS sequences. A, combined *LFY* and *NLY*; B, *LFY*; C, *NLY*. Numbers associated with branches are bootstrap percentages of ML and MP higher than 50% and Bayesian posterior probabilities greater than 0.90, respectively. *Taiwania cryptomerioides* and *Cunninghamia lanceolata* were used as outgroups.

The present study seems to supports the monophyly of Taxaceae *s.l.* that includes *Cephalotaxus* (Figs. 1, S1), which is consistent with the study of Leslie *et al.* [18] based on *rbc*L, *mat*K, 18S and *PHY*P. To confirm whether the topology is really constant, we further conducted phylogenetic analyses for the Cephalotaxaceae-Taxaceae lineage using two species (*Taiwania cryptomerioides* and *Cunninghamia lanceolata*) of its sister group Cupressaceae as outgroups. The results indicate that *Cephalotaxus* is strongly supported to be sister to Taxaceae based on either *LFY* or *LFY* + *NLY* CDS, but is nested within Taxaceae with a weak support based on *NLY* (Fig. 6). The inconsistent positions of Cephalotaxaceae in different analyses could be caused by LBA artifacts or insufficient resolution of the markers. Actually, the evolutionary relationship of Cephalotaxaceae and Taxaceae has been controversial for a long time. All molecular studies based on chloroplast and/or nuclear ribosomal DNA suggested a sister relationship between the two families [91]–[93], but many morphological studies supported the merge of them (see review by Ghimire and Heo [94]). A more broadly defined Taxaceae including Cephalotaxaceae has been suggested by Quinn *et al.* [95] based on *rbc*L and *mat*K sequence analyses, and by Ghimire and Heo [94] based on a cladistic analysis of morphological characters. Also, in the new gymnosperm classification scheme of Christenhusz *et al.* [2], Cephalotaxaceae was merged into Taxaceae, and this taxonomic treatment has been adopted by Lang *et al.* [96] in the revision of *Cephalotaxus*. As discussed above, more studies are still needed to resolve the relationship between Cephalotaxaceae and Taxaceae.

The systematic position of Gnetales has been debated for several decades, which involves six main hypotheses (see reviews by Braukmann *et al.* [8] and Wang and Ran [13]), i.e., anthophyte, gnetales-other seed plants, gnetales-other gymnosperms, Gnetifer, Gnecup and Gnepine [4], [5], [8], [9], [80], [97]–[101]. The last three hypotheses all support a close relationship between Gnetales and conifers. In particular, the Gnepine hypothesis (Gnetales sister to Pinaceae) is supported by more and more molecular phylogenetic studies after eliminating bias in data analyses (see review by Wang and Ran [13]), despite the fact that the Gnecup hypothesis (Gnetales sister to conifer II or cupressophytes) is still supported by a couple of recent phylogenomic studies using all chloroplast genes [9], [12]. According to the present study, the Gnetales has a close relationship with conifers, although it has not been resolved whether the Gnecup or Gnepine hypothesis is correct. In the trees generated from combined LFY and NLY CDS, Gnetales is strongly supported as sister to conifer II (Fig. 2A and 2C), which is corroborated by the SH and KH tests (Table S4). However, when excluding the third codon positions and using cycads as functional outgroups, the most popular Gnepine hypothesis is recovered with low support (Fig. 2D, Table S4). A similar phenomenon is also observed in Sciadopityaceae. When all CDS sequences are used, this family is moderately supported as sister to the Podocarpaceae-Araucariaceae clade (Fig. 2A and 2C), but when excluding the third codon positions it is revealed as sister to the Taxaceae-Cephalotaxaceae-Cupressaceae clade (Fig. 2B and 2D) as found in most previous studies [14], [16]–[18], [22]. The topological conflicts on phylogenetic positions of Gnetales and Sciadopityaceae may be attributed to LBA artifacts that could occur when the fast-evolving third codon positions are included in analyses. Zhong *et al.* [9] also found that the LBA artifacts and parallel changes could mislead the phylogenetic placement of Gnetales when using chloroplast genome data, and the removal of fast-evolving genes can effectively alleviate the LBA artifacts, thereby recovering a sister relationship between Gnetales and Pinaceae.

### Intergeneric relationships within gymnospermous families

The combined *LFY* and *NLY* CDS phylogeny provides a good resolution for intergeneric relationships within four families including Cupressaceae, Pinaceae, Taxaceae and Araucariaceae (Figs. 1, S1). The *LFY* + *NLY* phylogeny of Cupressaceae has been discussed in detail by Yang *et al.* [22]. For Pinaceae, all of the eleven genera form two strongly supported clades. One clade comprises *Cedrus*, *Pseudolarix*, and two pairs of sister genera, i.e., *Nothotsuga*-*Tsuga* and *Keteleeria*-*Abies*, while the other clade includes the sister genera *Pseudotsuga* and *Larix*, and the three closely related genera *Pinus*, *Cathaya* and *Picea* (Figs. 1, S1). The revealed intergeneric relationships are largely congruent with the finding of Wang *et al.* [23], and are generally consistent with the results of morphological and anatomical analyses (see review by Farjón [102]). However, Wang *et al.* [23] did not completely resolve the systematic position of *Cedrus*. According to the present study, *Cedrus* is sister to the *Nothotsuga*-*Tsuga*-*Pseudolarix*-*Keteleeria*-*Abies* clade (Figs. 1, S1), which is consistent with most recent molecular phylogenetic studies [18], [29]. Moreover, like Wang *et al.* [23], our study supports the monotypic genus *Cathaya* as sister to *Picea* (Figs. 1, S1), rather than to *Pinus* as suggested by Lin *et al.* [29]. In Taxaceae, *Torreya* is sister to *Amentotaxus*, and *Austrotaxus* is closely related to the sister genera *Pseudotaxus* and *Taxus* (Figs. 1, 6, S1), corroborating previous studies [18], [91]–[93]. For Araucariaceae, the previous *rbc*L gene analysis suggested a basal position of *Wollemia* in the family [103]. However, the present study supports *Wollemia* as sister to *Agathis* (Figs. 1, S1), consistent with more recent studies [18], [95], [104], [105].

The concatenated *LFY* and *NLY* CDS can not resolve some intergeneric relationships of cycads and Podocarpaceae very well (Figs. 1, S1). It is interesting that the addition of intron sequences can improve the resolution in Podocarpaceae but not in cycads (Figs. 3, 4), although divergence times of the cycad genera are similar to or longer than those of the Podocarpaceae genera (Fig. 5) [18], [25]. Consistent with most previous studies [24], [31], [32], [79], the phylogeny of cycads inferred from either CDS or CDS+Intron sequences of *LFY* and *NLY* supports the genus *Dioon* from tropical America as the basal-most lineage in Zamiaceae (Fig. 3), rather than sister to the *Bowenia*-*Ceratozamia*-*Stangeria*-*Microcycas*-*Zamia* clade in the *PHYP* tree constructed by Nagalingum *et al.* [25]. Actually, despite low support values in some clades, the present *LFY* + *NLY* gene tree is topologically very similar to the recently reconstructed phylogeny of cycads based on five single-copy nuclear genes [32]. For instance, the two genera *Zamia* and *Microcycas*, also from tropical America, have a sister relationship and form a clade sister to *Stangeria*, while the African *Encephalartos* and the Australian *Lepidozamia* form a clade sister to *Macrozamia* from Australia (Fig. 3). Moreover, our study also does not support the establishment of the family Boweniaceae or Stangeriaceae that was based on morphological analyses [106], [107], since the two genera *Bowenia* and *Stangeria* are nested within Zamiaceae and do not form a monophyletic clade (Fig. 3), as found in most previous molecular phylogenetic analyses [24], [25], [31], [79].

Compared to the CDS dataset, the CDS+Intron dataset provides a much better resolution for intergeneric relationships of Podocarpaceae (Fig. 4), a large family comprising 19 genera with a wide distribution in the tropics, especially in the Southern Hemisphere [108], [109]. Our study strongly supports a large clade comprising 11 genera, of which the Australian *Microcachrys* and the South American *Saxegothaea* diverged first, followed by the two Australian genera *Pherosphaera* and *Acmopyle*, and then the three genera *Dacrycarpus*, *Dacrydium* and *Falcatifolium* (all distributed in Asia and Australia) forming the ‘dacrydioid’ group sister to the ‘podocarpoid’ group that include *Retrophyllum*, *Nageia*, *Afrocarpus* and *Podocarpus*. In addition, we found a close relationship among the three Australian genera *Manoao*, *Lagarostrobos* and *Parasitaxus* and a sister relationship between *Prumnopitys* and *Sundacarpus* (Fig. 4B). This phylogeny of Podocarpaceae constructed from nuclear genes is topologically highly consistent with those inferred from plastid DNA fragments [110] and from a combined analysis of nrITS1, *NLY* intron 2 and *rbc*L sequences as well as anatomical and morphological data [30]. However, our nuclear gene phylogeny strongly supports two pairs of sister genera *Retrophyllum-Nageia* and *Afrocarpus-Podocarpus* (Fig. 4B). The genus *Phyllocladus* is nested within Podocarpaceae (Fig. 4), and thus the family status of Phyllocladaceae is not supported.

### Divergence times of gymnosperms

The divergence time estimation is very helpful to interpret the temporal evolution of organisms. Previous studies have provided divergence time estimates for different gymnospermous groups, such as Pinaceae [23], cycads [25], Podocarpaceae [110], Cupressaceae [21], [22], and conifers [18]. However, only Crisp and Cook [14] estimated divergence times of gymnosperms as a whole using molecular clock, and in their study many extant genera were not sampled.

Our present study provides divergence time estimates for gymnosperms based on a sampling of all extant families and genera (Fig. 5). The estimated crown ages of some groups such as Pinaceae, cycads and Podocarpaceae are approaching to those reported in previous studies [18], [25], [110]. However, the estimated crown age of Cupressaceae and divergence times of most genera of this family are a little younger than those reported in Yang *et al.* [22] and Mao *et al.* [21]. This could be attributed to the discrepancy of different dating methods and delineation of different fossil calibrations.

**Figure 7:**
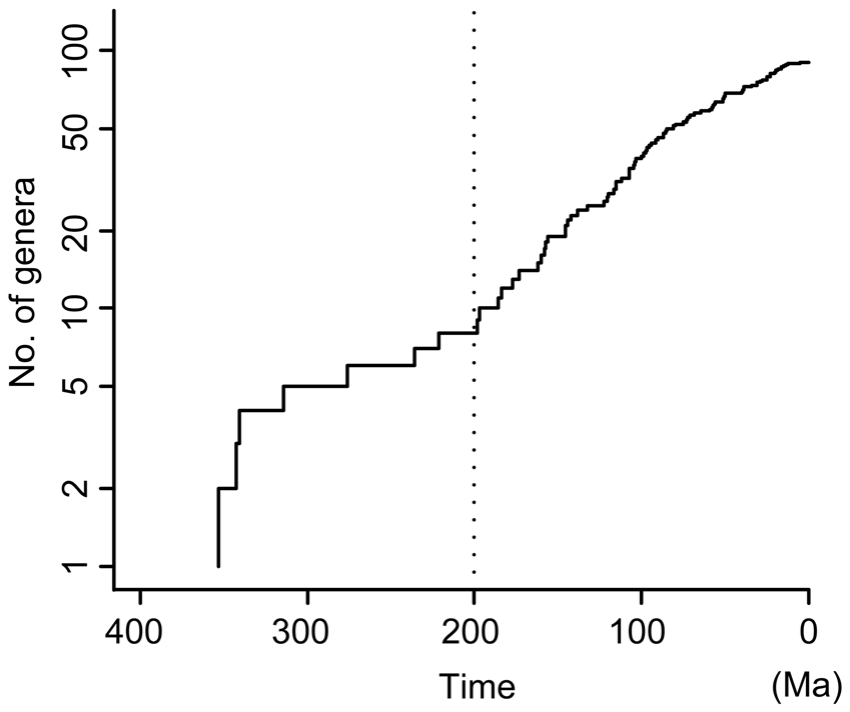
A lineage-through-time plot showing divergence time distribution of the gymnosperm genera. The divergence times was based on the median ages of the nodes from the BEAST analysis (see Fig. 5).

**Figure 8:**
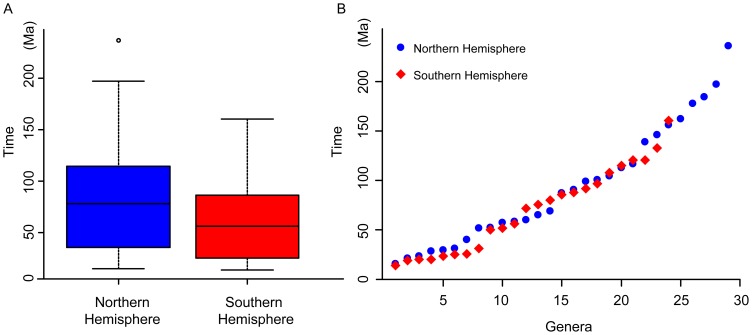
Comparison of divergence times of the coniferous genera between the Southern and Northern Hemispheres. A, boxplot comparison of all genera; B, dot plot comparison of each genus. The calculation of divergence times was based on the median ages of the nodes from the BEAST analysis as shown in Fig. 5.

Based on the molecular dating analysis, all of the extant five lineages of gymnosperms (cycads, ginkgos, cupressophytes, Pinaceae and gnetophytes) originated at least before 300 Ma (in the Carboniferous), but the crown ages of all families except Ginkgoaceae and Sciadopityaceae are younger than 200 Ma (Fig. 5), indicating that drastic extinctions occurred in the early evolution of gymnosperms, which might be caused by the two extreme cooling events in the Carboniferous and Triassic [111]. After 200 Ma, the divergence speed of genera is moderate when extinction is not considered (Fig. 7), although recent studies showed that the pulse of extinction and speciation in the Cenozoic, even in the late Tertiary, shaped today's species diversity of gymnosperms [14], [25]. Leslie *et al.* [18] found that lineages of conifers that diversified mainly in the Southern Hemisphere show a significantly older distribution of divergence ages than their counterparts in the Northern Hemisphere. However, interestingly, we found that extant coniferous genera in the Northern Hemisphere are older than those in the Southern Hemisphere on average (Fig. 8A). In fact, if excluding the several genera that originated before 150 Ma, the distribution of divergence ages of the remaining genera is very similar between the two hemispheres (Fig. 8B). Of great interest is to investigate why more ancient genera survive in the Northern Hemisphere than in the Southern Hemisphere. Moreover, to get a more accurate estimation of the divergence times and a solid reconstruction of the evolutionary dynamics of gymnosperms, more nuclear genes or genome sequences should be used in future studies, and more reliable fossils are needed to be found.

## Supporting Information

Figure S1 **The ML and BI trees of gymnosperms constructed from combined** ***LFY*** **and** ***NLY*** **sequences.** Numbers associated with branches are bootstrap percentages of ML higher than 50% and Bayesian posterior probabilities greater than 0.90, respectively. A, ML tree from the CDS sequences with *Angiopteris lygodiifolia* as outgroup; B, BI tree from the CDS sequences with *Angiopteris lygodiifolia* as outgroup; C, ML tree from the 1^st^+2^nd^ codon positions with *Angiopteris lygodiifolia* as outgroup; D, BI tree from the 1^st^+2^nd^ codon positions with *Angiopteris lygodiifolia* as outgroup; E, BI tree from the CDS sequences with cycads as functional outgroups; F, ML tree from the 1^st^+2^nd^ codon positions with cycads as functional outgroups; G, BI tree from the 1^st^+2^nd^ codon positions with cycads as functional outgroups.
(PDF)

Table S1 Sources of materials.
(DOC)

Table S2 Primers used for the PCR amplification and sequencing.
(DOC)

Table S3 The eleven calibration points used in divergence time estimation for gymnosperms. All constraints were given lognormal prior distributions, where the minimum age was set by the age of the fossil constraint and 95% confidence interval of the probability distribution extending 20 or 40 million years earlier than the minimum age.
(DOC)

Table S4 Results of the Shimodaira-Hasegawa (SH) test and the Kishino-Hasegawa (KH) test.
(DOC)

Table S5 Ages of selected clades.
(DOC)

## References

[1] The Angiosperm Phylogeny Group (2009) An update of the Angiosperm Phylogeny Group classification for the orders and families of flowering plants: APG III. Bot J Linn Soc 161: 105–121.

[2] Christenhusz MJM, Reveal JL, Farjón A, Gardner MF, Mill RR, et al (2011) A new classification and linear sequence of extant gymnosperms. Phytotaxa 19: 55–70.

[3] Winter KU, Bec ker A, Munster T, Kim JT, Saedler H, et al (1999) MADS-box genes reveal that gnetophytes are more closely related to conifers than to flowering plants. Proc Natl Acad Sci U S A 96: 7342–7347. 10377416 10.1073/pnas.96.13.7342 PMC22087

[4] Chaw SM, Parkinson CL, Cheng YC, Vincent TM, Palmer JD (2000) Seed plant phylogeny inferred from all three plant genomes: Monophyly of extant gymnosperms and origin of Gnetales from conifers. Proc Natl Acad Sci U S A 97: 4086–4091. 10760277 10.1073/pnas.97.8.4086 PMC18157

[5] Bowe LM, Coat G, dePamphilis CW (2000) Phylogeny of seed plants based on all three genomic compartments: Extant gymnosperms are monophyletic and Gnetales' closest relatives are conifers. Proc Natl Acad Sci U S A 97: 4092–4097. 10760278 10.1073/pnas.97.8.4092 PMC18159

[6] Mundry M, Stutzel T (2004) Morphogenesis of the reproductive shoots of *Welwitschia mirabilis* and *Ephedra distachya* (Gnetales), and its evolutionary implications. Org Divers Evol 4: 91–108.

[7] Hajibabaei M, Xia J, Drouin G (2006) Seed plant phylogeny: Gnetophytes are derived conifers and a sister group to Pinaceae. Mol Phylogenet Evol 40: 208–217. 16621615 10.1016/j.ympev.2006.03.006

[8] Braukmann TWA, Kuzmina M, Stefanović S (2009) Loss of all plastid *ndh* genes in Gnetales and conifers: extent and evolutionary significance for the seed plant phylogeny. Curr Genet 55: 323–337. 19449185 10.1007/s00294-009-0249-7

[9] Zhong B, Yonezawa T, Zhong Y, Hasegawa M (2010) The position of gnetales among seed plants: overcoming pitfalls of chloroplast phylogenomics. Mol Biol Evol 27: 2855–2863. 20601411 10.1093/molbev/msq170

[10] Wu CS, Lin CP, Hsu CY, Wang RJ, Chaw SM (2011) Comparative chloroplast genomes of Pinaceae: insights into the mechanism of diversified genomic organizations. Genome Biol Evol 3: 309–319. 21402866 10.1093/gbe/evr026 PMC5654405

[11] Xi Z, Rest JS, Davis CC (2013) Phylogenomics and coalescent analyses resolve extant seed plant relationships. PLoS One 8: e80870. 24278335 10.1371/journal.pone.0080870 PMC3836751

[12] Ruhfel B, Gitzendanner M, Soltis P, Soltis D, Burleigh J (2014) From algae to angiosperms-inferring the phylogeny of green plants (Viridiplantae) from 360 plastid genomes. BMC Evol Biol 14: 23. 24533922 10.1186/1471-2148-14-23 PMC3933183

[13] Wang X-Q, Ran J-H (2014) Evolution and biogeography of gymnosperms. Mol Phylogenet Evol 75: 24–40. 24565948 10.1016/j.ympev.2014.02.005

[14] Crisp MD, Cook LG (2011) Cenozoic extinctions account for the low diversity of extant gymnosperms compared with angiosperms. New Phytol 192: 997–1009. 21895664 10.1111/j.1469-8137.2011.03862.x

[15] Chaw SM, Zharkikh A, Sung HM, Lau TC, Li WH (1997) Molecular phylogeny of extant gymnosperms and seed plant evolution: analysis of nuclear 18S rRNA sequences. Mol Biol Evol 14: 56–68. 9000754 10.1093/oxfordjournals.molbev.a025702

[16] Ran J-H, Gao H, Wang X-Q (2010) Fast evolution of the retroprocessed mitochondrial *rps3* gene in Conifer II and further evidence for the phylogeny of gymnosperms. Mol Phylogenet Evol 54: 136–149. 19761858 10.1016/j.ympev.2009.09.011

[17] Rai HS, Reeves PA, Peakall R, Olmstead RG, Graham SW (2008) Inference of higher-order conifer relationships from a multi-locus plastid data set. Botany 86: 658–669.

[18] Leslie AB, Beaulieu JM, Rai HS, Crane PR, Donoghue MJ, et al (2012) Hemisphere-scale differences in conifer evolutionary dynamics. Proc Natl Acad Sci U S A 109: 16217–16221. 22988083 10.1073/pnas.1213621109 PMC3479534

[19] Gadek PA, Alpers DL, Heslewood MM, Quinn CJ (2000) Relationships within Cupressaceae *sensu lato*: A combined morphological and molecular approach. Am J Bot 87: 1044–1057. 10898782

[20] Kusumi J, Tsumura Y, Yoshimaru H, Tachida H (2000) Phylogenetic relationships in Taxodiaceae and Cupressaceae *sensu stricto* based on *mat*K gene, *chl*L gene, *trn*L-*trn*F IGS region, and *trn*L intron sequences. Am J Bot 87: 1480–1488. 11034923

[21] Mao K, Milne RI, Zhang L, Peng Y, Liu J, et al (2012) Distribution of living Cupressaceae reflects the breakup of Pangea. Proc Natl Acad Sci U S A 109: 7793–7798. 22550176 10.1073/pnas.1114319109 PMC3356613

[22] Yang Z-Y, Ran J-H, Wang X-Q (2012) Three genome-based phylogeny of Cupressaceae *s.l.*: Further evidence for the evolution of gymnosperms and Southern Hemisphere biogeography. Mol Phylogenet Evol 64: 452–470. 22609823 10.1016/j.ympev.2012.05.004

[23] Wang X-Q, Tank DC, Sang T (2000) Phylogeny and divergence times in Pinaceae: evidence from three genomes. Mol Biol Evol 17: 773–781. 10779538 10.1093/oxfordjournals.molbev.a026356

[24] Zgurski JM, Rai HS, Fai QM, Bogler DJ, Francisco-Ortega J, et al (2008) How well do we understand the overall backbone of cycad phylogeny? New insights from a large, multigene plastid data set. Mol Phylogenet Evol 47: 1232–1237. 18424186 10.1016/j.ympev.2008.03.002

[25] Nagalingum NS, Marshall CR, Quental TB, Rai HS, Little DP, et al (2011) Recent synchronous radiation of a living fossil. Science 334: 796–799. 22021670 10.1126/science.1209926

[26] Wu CS, Chaw SM, Huang YY (2013) Chloroplast phylogenomics indicates that *Ginkgo biloba* is sister to cycads. Genome Biol Evol 5: 243–254. 23315384 10.1093/gbe/evt001 PMC3595029

[27] Lee EK, Cibrian-Jaramillo A, Kolokotronis SO, Katari MS, Stamatakis A, et al (2011) A functional phylogenomic view of the seed plants. PLoS Genet 7: e1002411. 22194700 10.1371/journal.pgen.1002411 PMC3240601

[28] Mogensen HL (1996) The hows and whys of cytoplasmic inheritance in seed plants. Am J Bot 83: 383–404.

[29] Lin CP, Huang JP, Wu CS, Hsu CY, Chaw SM (2010) Comparative chloroplast genomics reveals the evolution of Pinaceae genera and subfamilies. Genome Biol Evol 2: 504–517. 20651328 10.1093/gbe/evq036 PMC2997556

[30] Knopf P, Schulz C, Little DP, Stutzel T, Stevenson DW (2012) Relationships within Podocarpaceae based on DNA sequence, anatomical, morphological, and biogeographical data. Cladistics 28: 271–299. 10.1111/j.1096-0031.2011.00381.x 34872191

[31] Chaw SM, Walters TW, Chang CC, Hu SH, Chen SH (2005) A phylogeny of cycads (Cycadales) inferred from chloroplast *matK* gene, *trnK* intron, and nuclear rDNA ITS region. Mol Phylogenet Evol 37: 214–234. 16182153 10.1016/j.ympev.2005.01.006

[32] Salas-Leiva DE, Meerow AW, Calonje M, Griffith MP, Francisco-Ortega J, et al (2013) Phylogeny of the cycads based on multiple single-copy nuclear genes: congruence of concatenated parsimony, likelihood and species tree inference methods. Ann Bot 112: 1263–1278. 23997230 10.1093/aob/mct192 PMC3806525

[33] Zhang N, Zeng L, Shan H, Ma H (2012) Highly conserved low-copy nuclear genes as effective markers for phylogenetic analyses in angiosperms. New Phytol 195: 923–937. 22783877 10.1111/j.1469-8137.2012.04212.x

[34] Nystedt B, Street NR, Wetterbom A, Zuccolo A, Lin YC, et al (2013) The Norway spruce genome sequence and conifer genome evolution. Nature 497: 579–584. 23698360 10.1038/nature12211

[35] Vazquez-Lobo A, Carlsbecker A, Vergara-Silva F, Alvarez-Buylla ER, Pinero D, et al (2007) Characterization of the expression patterns of *LEAFY/FLORICAULA* and *NEEDLY* orthologs in female and male cones of the conifer genera *Picea*, *Podocarpus*, and *Taxus*: implications for current evo-devo hypotheses for gymnosperms. Evol Dev 9: 446–459. 17845516 10.1111/j.1525-142X.2007.00182.x

[36] Moyroud E, Kusters E, Monniaux M, Koes R, Parcy F (2010) *LEAFY* blossoms. Trends Plant Sci 15: 346–352. 20413341 10.1016/j.tplants.2010.03.007

[37] Syring J, Farrell K, Businský R, Cronn R, Liston A (2007) Widespread genealogical nonmonophyly in species of *Pinus* subgenus *Strobus* . Syst. Biol 56: 163–181. 17454973 10.1080/10635150701258787

[38] Rogers SO, Bendich AJ (1985) Extraction of DNA from milligram amounts of fresh, herbarium and mummified plant-tissues. Plant Mol Biol 5: 69–76. 24306565 10.1007/BF00020088

[39] Thompson JD, Gibson TJ, Plewniak F, Jeanmougin F, Higgins DG (1997) The CLUSTAL_X windows interface: flexible strategies for multiple sequence alignment aided by quality analysis tools. Nucl Acids Res 25: 4876–4882. 9396791 10.1093/nar/25.24.4876 PMC147148

[40] Tamura K, Peterson D, Peterson N, Stecher G, Nei M, et al (2011) MEGA5: Molecular evolutionary genetics analysis using maximum likelihood, evolutionary distance, and maximum parsimony methods. Mol Biol Evol 28: 2731–2739. 21546353 10.1093/molbev/msr121 PMC3203626

[41] Hall TA (1999) BioEdit: a user-friendly biological sequence alignment editor and analysis program for Windows 95/98/NT. Nucl Acids Symp Ser 40: 95–98.

[42] Xia X, Xie Z (2001) DAMBE: software package for data analysis in molecular biology and evolution. J Hered 92: 371–373. 11535656 10.1093/jhered/92.4.371

[43] Farris JS, Kallersjo M, Kluge AG, Bult C (1994) Testing significance of incongruence. Cladistics 10: 315–319.

[44] Swofford DL (2002) Phylogenetic Analysis Using Parsimony (* and other methods), Version 4. Sinauer, Sunderland, MA.

[45] Leigh JW, Susko E, Baumgartner M, Roger AJ (2008) Testing congruence in phylogenomic analysis. Syst Biol 57: 104–115. 18288620 10.1080/10635150801910436

[46] Campbell V, Legendre P, Lapointe F-J (2009) Assessing congruence among ultrametric distance matrices. J Classif 26: 103–117.

[47] Felsenstein J (1985) Confidence-limits on phylogenies - an approach using the Bootstrap. Evolution 39: 783–791. 28561359 10.1111/j.1558-5646.1985.tb00420.x

[48] Darriba D, Taboada GL, Doallo R, Posada D (2012) jModelTest 2: more models, new heuristics and parallel computing. Nat Methods 9: 772. 10.1038/nmeth.2109 PMC4594756 22847109

[49] Nylander JAA (2004) MrModeltest v2. Program distributed by the author: Evolutionary Biology Centre, Uppsala Univ., Uppsala, Sweden.

[50] Guindon S, Gascuel O (2003) A simple, fast, and accurate algorithm to estimate large phylogenies by maximum likelihood. Syst Biol 52: 696–704. 14530136 10.1080/10635150390235520

[51] Ronquist F, Huelsenbeck JP (2003) MrBayes 3: Bayesian phylogenetic inference under mixed models. Bioinformatics 19: 1572–1574. 12912839 10.1093/bioinformatics/btg180

[52] Shimodaira H, Hasegawa M (1999) Multiple comparisons of log-likelihoods with applications to phylogenetic inference. Mol Biol Evol 16: 1114–1116.

[53] Kishino H, Hasegawa M (1989) Evaluation of the maximum likelihood estimate of the evolutionary tree topologies from DNA sequence data, and the branching order in hominoidea. J Mol Evol 29: 170–179. 2509717 10.1007/BF02100115

[54] Drummond AJ, Suchard MA, Xie D, Rambaut A (2012) Bayesian phylogenetics with BEAUti and the BEAST 1.7. Mol Biol Evol 29: 1969–1973. 22367748 10.1093/molbev/mss075 PMC3408070

[55] Sauquet H, Ho SY, Gandolfo MA, Jordan GJ, Wilf P, et al (2012) Testing the impact of calibration on molecular divergence times using a fossil-rich group: the case of Nothofagus (Fagales). Syst Biol 61: 289–313. 22201158 10.1093/sysbio/syr116

[56] Magallón S, Hilu KW, Quandt D (2013) Land plant evolutionary timeline: Gene effects are secondary to fossil constraints in relaxed clock estimation of age and substitution rates. Am J Bot 100: 556–573. 23445823 10.3732/ajb.1200416

[57] Ravn RL, Swade JW, Howes RR, Gregory JL, Anderson RR, et al (1984) Stratigraphy of the Cherokee Group and revision of Pennsylvanian stratigraphic nomenclature in Iowa. Iowa Geological Survey Technical Information Series 12: 1–76.

[58] Rothwell GW, Scheckler SE, Gillespie WH (1989) *Elkinsia* gen. nov., a late Devonian gymnosperm with cupulate ovules. Bot Gaz 150: 170–189.

[59] Peppers RA (1996) Palynological correlation of major Pennsylvanian (Middle and Upper Carboniferous) chronostratigraphic boundaries in the Illinois and other coal basins. Geol Soc Amer Mem 188: 1–111.

[60] Hill RS (1980) Three new Eocene cycads from eastern Australia. Aust J Bot 28: 105.

[61] Klymiuk AA, Stockey RA (2012) A Lower Cretaceous (Valanginian) seed cone provides the earliest fossil record for *Picea* (Pinaceae). Am J Bot 99: 1069–1082. 22623610 10.3732/ajb.1100568

[62] Krassilov VA (1982) Early Cretaceous flora of Mongolia. Palaeontolographica B 181: 1–43.

[63] Krassilov VA (1986) New floral structure from the Lower Cretaceous of Lake Bakal area. Rev Paleobot Palinol 47: 9–16.

[64] Harris TM (1979) The Yorkshire Jurassic Flora. V. Coniferales. British Museum of Natural History, London.

[65] Hill RS, Merrifield HE (1993) An early Tertiary macroflora from West Dale, southwestern Australia. Alcheringa 17: 285–326.

[66] Florin R (1958) On Jurassic taxads and conifers from north-western Europe and Eastern Greenland. Acta Hort Berg 17: 257–402.

[67] Penny JS (1947) Studies on the conifers of the magothy flora. Am J Bot 34: 281–296.

[68] Ma Q-W, Li F-L, Li C-S (2005) The coast redwoods (*Sequoia*, Taxodiaceae) from the Eocene of Heilongjiang and the Miocene of Yunnan, China. Rev Palaeobot Palynol 135: 117–129.

[69] Aulenback KR, LePage BA (1998) *Taxodium wallisii* sp. nov.: first occurrence of *Taxodium* from the Upper Cretaceous. Int J Plant Sci 159: 367–390.

[70] Miller CN (1977) Mesozoic conifers. Bot Rev 43: 217–280.

[71] McIver EE (2001) Cretaceous *Widdringtonia* Endl. (Cupressaceae) from North America. Int J Plant Sci 162: 937–961.

[72] Kvacek Z (2002) A new juniper from the Palaeogene of Central Europe. Fedd Repert 113: 492–502.

[73] Felsenstein J (1978) Cases in which parsimony or compatibility methods will be positively misleading. Syst Zool 27: 401–410.

[74] Hendy MD, Penny D (1989) A framework for the quantitative study of evolutionary trees. Syst Zool 38: 297–309.

[75] Huelsenbeck JP (1995) Performance of phylogenetic methods in simulation. Syst Biol 44: 17–48.

[76] Conran JG, Wood GM, Martin PG, Dowd JM, Quinn CJ, et al (2000) Generic relationships within and between the gymnosperm families Podocarpaceae and Phyllocladaceae based on an analysis of the chloroplast gene *rbcL* . Aust J Bot 48: 715–724.

[77] Kelch D (1998) Phylogeny of Podocarpaceae: comparison of evidence from morphology and 18S rDNA. Am J Bot 85: 986. 21684982

[78] Hill KD, Chase MW, Stevenson DW, Hills HG, Schutzman B (2003) The families and genera of cycads: A molecular phylogenetic analysis of cycadophyta based on nuclear and plastid DNA sequences. Int J Plant Sci 164: 933–948.

[79] Rai HS, O'Brien HE, Reeves PA, Olmstead RG, Graham SW (2003) Inference of higher-order relationships in the cycads from a large chloroplast data set. Mol Phylogenet Evol 29: 350–359. 13678689 10.1016/s1055-7903(03)00131-3

[80] Frohlich MW, Parker DS (2000) The mostly male theory of flower evolutionary origins: from genes to fossils. Syst Bot 25: 155–170.

[81] Frohlich MW (2003) An evolutionary scenario for the origin of flowers. Nat Rev Genet 4: 559–566. 12838347 10.1038/nrg1114

[82] Mellerowicz EJ, Horgan K, Walden A, Coker A, Walter C (1998) *PRFLL* - a *Pinus radiata* homologue of *FLORICAULA* and *LEAFY* is expressed in buds containing vegetative shoot and undifferentiated male cone primordia. Planta 206: 619–629. 9821691 10.1007/s004250050440

[83] Mouradov A, Glassick T, Hamdorf B, Murphy L, Fowler B, et al (1998) *NEEDLY*, a *Pinus radiata* ortholog of *FLORICAULA/LEAFY* genes, expressed in both reproductive and vegetative meristems. Proc Natl Acad Sci U S A 95: 6537–6542. 9601002 10.1073/pnas.95.11.6537 PMC27861

[84] Shindo S, Sakakibara K, Sano R, Ueda K, Hasebe M (2001) Characterization of a *FLORICAULA/LEAFY* homologue of *Gnetum parvifolium* and its implications for the evolution of reproductive organs in seed plants. Int J Plant Sci 162: 1199–1209.

[85] Carlsbecker A, Tandre K, Johanson U, Englund M, Engström P (2004) The MADS-box gene *DAL1* is a potential mediator of the juvenile-to-adult transition in Norway spruce (*Picea abies*). Plant J 40: 546–557. 15500470 10.1111/j.1365-313X.2004.02226.x

[86] Dornelas MC, Rodriguez APM (2005) A *FLORICAULA/LEAFY* gene homolog is preferentially expressed in developing female cones of the tropical pine *Pinus caribaea* var. *caribaea* . Genet Mol Biol 28: 299–307.

[87] Shiokawa T, Yamada S, Futamura N, Osanai K, Murasugi D, et al (2008) Isolation and functional analysis of the *CjNdly* gene, a homolog in *Cryptomeria japonica* of *FLORICAULA/LEAFY* genes. Tree Physiol 28: 21–28. 17938110 10.1093/treephys/28.1.21

[88] Won H, Renner SS (2006) Dating dispersal and radiation in the gymnosperm *Gnetum* (Gnetales) - Clock calibration when outgroup relationships are uncertain. Syst Biol 55: 610–622. 16969937 10.1080/10635150600812619

[89] Peng D, Wang X-Q (2008) Reticulate evolution in *Thuja* inferred from multiple gene sequences: implications for the study of biogeographical disjunction between eastern Asia and North America. Mol Phylogenet Evol 47: 1190–1202. 18346917 10.1016/j.ympev.2008.02.001

[90] Wei X-X, Yang Z-Y, Li Y, Wang X-Q (2010) Molecular phylogeny and biogeography of *Pseudotsuga* (Pinaceae): insights into the floristic relationship between Taiwan and its adjacent areas. Mol Phylogenet Evol 55: 776–785. 20214996 10.1016/j.ympev.2010.03.007

[91] Cheng Y, Nicolson RG, Tripp K, Chaw SM (2000) Phylogeny of Taxaceae and Cephalotaxaceae genera inferred from chloroplast *mat*K gene and nuclear rDNA ITS region. Mol Phylogenet Evol 14: 353–365. 10712841 10.1006/mpev.1999.0710

[92] Wang X-Q, Shu Y-Q (2000) Chloroplast *mat*K gene phylogeny of Taxaceae and Cephalotaxaceae, with additional reference to the systematic position of *Nageia* . Acta Phytotax Sin 38: 201–210.

[93] Hao DC, Xiao PG, Huang BL, Ge GB, Yang L (2008) Interspecific relationships and origins of Taxaceae and Cephalotaxaceae revealed by partitioned Bayesian analyses of chloroplast and nuclear DNA sequences. Plant Syst Evol 276: 89–104.

[94] Ghimire B, Heo K (2014) Cladistic analysis of Taxaceae *s.l.* . Plant Syst Evol 300: 217–223.

[95] Quinn CJ, Price RA, Gadek PA (2002) Familial concepts and relationships in the conifers based on *rbc*L and *mat*K sequence comparisons. Kew Bull 57 513–531.

[96] Lang XD, Su JR, Lu SG, Zhang ZJ (2013) A taxonomic revision of the genus *Cephalotaxus* (Taxaceae). Phytotaxa 84: 1–24.

[97] Donoghue MJ, Doyle JA (2000) Seed plant phylogeny: Demise of the anthophyte hypothesis? Curr Biol 10: R106–109. 10679315 10.1016/s0960-9822(00)00304-3

[98] Doyle JA, Donoghue MJ (1986) Seed plant phylogeny and the origin of angiosperms - an experimental cladistic approach. Bot Rev 52: 321–431.

[99] Schmidt M, Schneider-Poetsch HA (2002) The evolution of gymnosperms redrawn by phytochrome genes: the Gnetatae appear at the base of the gymnosperms. J Mol Evol 54: 715–724. 12029353 10.1007/s00239-001-0042-9

[100] Burleigh JG, Mathews S (2007) Assessing among-locus variation in the inference of seed plant phylogeny. Int J Plant Sci 168: 111–124.

[101] Zhong B, Deusch O, Goremykin VV, Penny D, Biggs PJ, et al (2011) Systematic error in seed plant phylogenomics. Genome Biol Evol 3: 1340–1348. 22016337 10.1093/gbe/evr105 PMC3237385

[102] Farjón A (1990) Pinaceae: Drawings and Descriptions of the Genera *Abies*, *Cedrus*, *Pseudolarix*, *Keteleeria*, *Nothotsuga*, *Tsuga*, *Cathaya*, *Pseudotsuga*, *Larix* and *Picea*. Königstein, Germany: Koeltz Scientific Books.

[103] Setoguchi H, Osawa TA, Pintaud JC, Jaffre T, Veillon JM (1998) Phylogenetic relationships within Araucariaceae based on *rbc*L gene sequences. Am J Bot 85: 1507–1516. 21680310

[104] Liu N, Zhu Y, Wei ZX, Chen J, Wang QB, et al (2009) Phylogenetic relationships and divergence times of the family Araucariaceae based on the DNA sequences of eight genes. Chinese Sci Bull 54: 2648–2655.

[105] Escapa IH, Catalano SA (2013) Phylogenetic analysis of Araucariaceae: Integrating molecules, morphology, and fossils. Int J Plant Sci 174: 1153–1170.

[106] Stevenson DW (1992) A formal classification of the extant cycads. Brittonia 44: 220–223.

[107] Stevenson DW (1990) Morphology and systematics of the Cycadales. Mem NY Bot Gard 57: 8–55.

[108] Eckenwalder JE (2009) Conifers of the World: the Complete Reference. London: Timber Press.

[109] Farjón A (2010) A Handbook of the World Conifers. Vol. 1, 2. Leiden: Brill Press.

[110] Biffin E, Brodribb TJ, Hill RS, Thomas P, Lowe AJ (2012) Leaf evolution in Southern Hemisphere conifers tracks the angiosperm ecological radiation. Proc Roy Soc B-Biol Sci 279: 341–348. 10.1098/rspb.2011.0559 PMC3223667 21653584

[111] Royer DL, Berner RA, Montañez IP, Tabor NJ, Beerling DJ (2004) CO2 as a primary driver of Phanerozoic climate. GSA Today 14: 4.

