# Supplementary material for: Phylogeny and Divergence Times of Gymnosperms Inferred from Single-Copy Nuclear Genes
Source: PLoS One. 2014 Sep 15;9(9):e107679. doi: 10.1371/journal.pone.0107679 (PMC4164646; doi:10.1371/journal.pone.0107679)
Supplement: Figure S1 — The ML and BI trees of gymnosperms constructed from combined LFY and NLY sequences. Numbers associated with branches are bootstrap percentages of ML higher than 50% and Bayesian posterior probabilities greater than 0.90, respectively. A, ML tree from the CDS sequences with Angiopteris lygodiifolia as outgroup; B, BI tree from the CDS sequences with Angiopteris lygodiifolia as outgroup; C, ML tree from the 1st+2nd codon positions with Angiopteris lygodiifolia as outgroup; D, BI tree from the 1st+2nd codon positions with Angiopteris lygodiifolia as outgroup; E, BI tree from the CDS sequences with cycads as functional outgroups; F, ML tree from the 1st+2nd codon positions with cycads as functional outgroups; G, BI tree from the 1st+2nd codon positions with cycads as functional outgroups. (PDF) [file pone.0107679.s001.pdf]

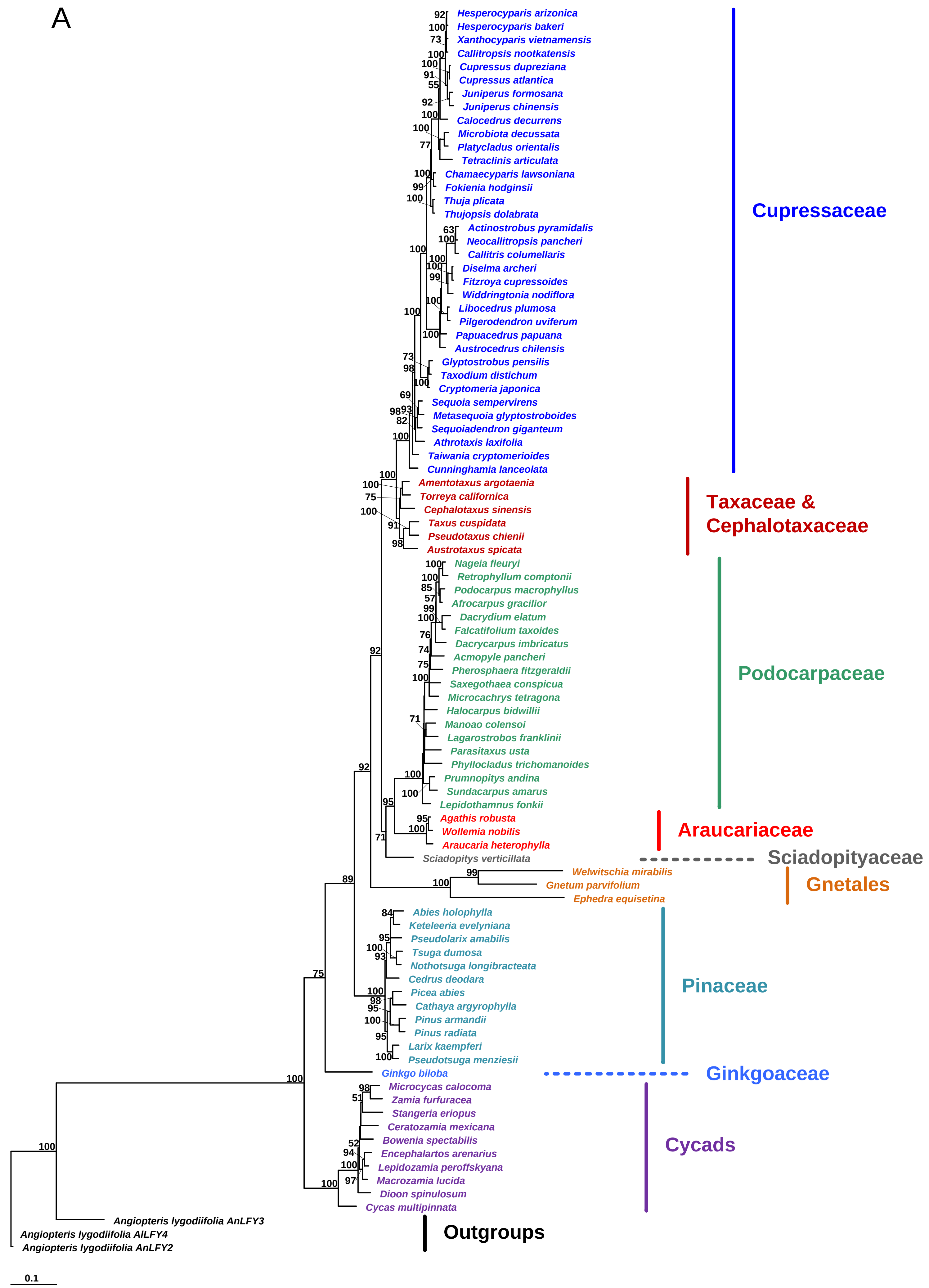
A
Hesperocyparis arizonica
Hesperocyparis bakeri
Xanthocyparis vietnamensis
Callitropsis nootkatensis
Cupressus dupreziana
Cupressus atlantica
Juniperus formosana
Juniperus chinensis
Calocedrus decurrens
Microbiota decussata
Platycladus orientalis
Tetraclinis articulata
Chamaecyparis lawsoniana
Fokienia hodginsii
Thuja plicata
Thujopsis dolabrata
Actinostrobus pyramidalis
Neocallitropsis pancheri
Callitris columellaris
Diselma archeri
Fitzroya cupressoides
Widdringtonia nodiflora
Libocedrus plumosa
Pilgerodendron uviferum
Papuacedrus papuana
Austrocedrus chilensis
Glyptostrobus pensilis
Taxodium distichum
Cryptomeria japonica
Sequoia sempervirens
Metasequoia glyptostroboides
Sequoiadendron giganteum
Athrotaxis laxifolia
Taiwania cryptomerioides
Cunninghamia lanceolata
Cupressaceae
Amentotaxus argotaenia
Torreya californica
Cephalotaxus sinensis
Taxus cuspidata
Pseudotaxus chienii
Austrotaxus spicata
Taxaceae &
Cephalotaxaceae
Nageia fleuryi
Retrophyllum comptonii
Podocarpus macrophyllus
Afrocarpus gracilior
Dacrydium elatum
Falcatifolium taxoides
Dacrycarpus imbricatus
Acmopyle pancheri
Pherosphaera fitzgeraldii
Saxegothaea conspicua
Microcachrys tetragona
Halocarpus bidwillii
Manoao colensoi
Lagarostrobos franklinii
Parasitaxus usta
Phyllocladus trichomanoides
Prumnopitys andina
Sundacarpus amarus
Lepidothamnus fonkii
Podocarpaceae
Agathis robusta
Wollemia nobilis
Araucaria heterophylla
Araucariaceae
Sciadopitys verticillata
Sciadopityaceae
Welwitschia mirabilis
Gnetum parvifolium
Ephedra equisetina
Gnetales
Abies holophylla
Keteleeria evelyniana
Pseudolarix amabilis
Tsuga dumosa
Nothotsuga longibracteata
Cedrus deodara
Picea abies
Cathaya argyrophylla
Pinus armandii
Pinus radiata
Larix kaempferi
Pseudotsuga menziesii
Pinaceae
Ginkgo biloba
Ginkgoaceae
Microcycas calocoma
Zamia furfuracea
Stangeria eriopus
Ceratozamia mexicana
Bowenia spectabilis
Encephalartos arenarius
Lepidozamia peroffskyana
Macrozamia lucida
Dioon spinulosum
Cycas multipinnata
Cycads
Angiopteris lygodiifolia AnLFY3
Angiopteris lygodiifolia AlLFY4
Angiopteris lygodiifolia AnLFY2
Outgroups
0.1

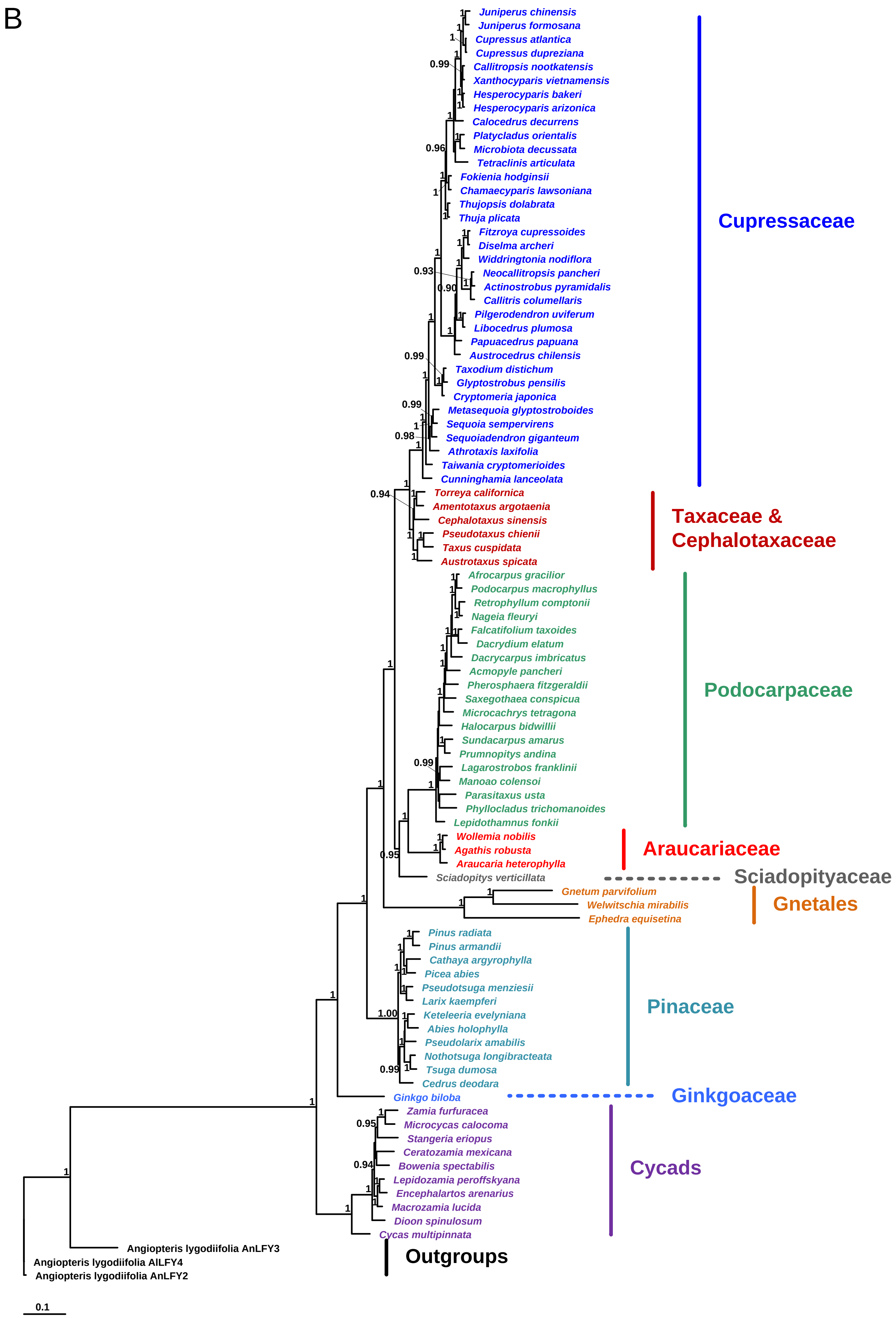
B
Juniperus chinensis
Juniperus formosana
Cupressus atlantica
Cupressus dupreziana
Callitropsis nootkatensis
Xanthocyparis vietnamensis
Hesperocyparis bakeri
Hesperocyparis arizonica
Calocedrus decurrens
Platycladus orientalis
Microbiota decussata
Tetraclinis articulata
Fokienia hodginsii
Chamaecyparis lawsoniana
Thujopsis dolabrata
Thuja plicata
Fitzroya cupressoides
Diselma archeri
Widdringtonia nodiflora
Neocallitropsis pancheri
Actinostrobus pyramidalis
Callitris columellaris
Pilgerodendron uviferum
Libocedrus plumosa
Papuacedrus papuana
Austrocedrus chilensis
Taxodium distichum
Glyptostrobus pensilis
Cryptomeria japonica
Metasequoia glyptostroboides
Sequoia sempervirens
Sequoiadendron giganteum
Athrotaxis laxifolia
Taiwania cryptomerioides
Cunninghamia lanceolata
Torreya californica
Amentotaxus argotaenia
Cephalotaxus sinensis
Pseudotaxus chienii
Taxus cuspidata
Austrotaxus spicata
Afrocarpus gracilior
Podocarpus macrophyllus
Retrophyllum comptonii
Nageia fleuryi
Falcatifolium taxoides
Dacrydium elatum
Dacrycarpus imbricatus
Acmopyle pancheri
Pherosphaera fitzgeraldii
Saxegothaea conspicua
Microcachrys tetragona
Halocarpus bidwillii
Sundacarpus amarus
Prumnopitys andina
Lagarostrobos franklinii
Manoao colensoi
Parasitaxus usta
Phyllocladus trichomanoides
Lepidothamnus fonkii
Wollemia nobilis
Agathis robusta
Araucaria heterophylla
Sciadopitys verticillata
Gnetum parvifolium
Welwitschia mirabilis
Ephedra equisetina
Pinus radiata
Pinus armandii
Cathaya argyrophylla
Picea abies
Pseudotsuga menziesii
Larix kaempferi
Keteleeria evelyniana
Abies holophylla
Pseudolarix amabilis
Nothotsuga longibracteata
Tsuga dumosa
Cedrus deodara
Ginkgo biloba
Zamia furfuracea
Microcycas calocoma
Stangeria eriopus
Ceratozamia mexicana
Bowenia spectabilis
Lepidozamia peroffskyana
Encephalartos arenarius
Macrozamia lucida
Dioon spinulosum
Cycas multipinnata
Angiopteris lygodiifolia AnLFY3
Angiopteris lygodiifolia AlLFY4
Angiopteris lygodiifolia AnLFY2
Cupressaceae
Taxaceae &
Cephalotaxaceae
Podocarpaceae
Araucariaceae
Sciadopityaceae
Gnetales
Pinaceae
Ginkgoaceae
Cycads
Outgroups
0.1

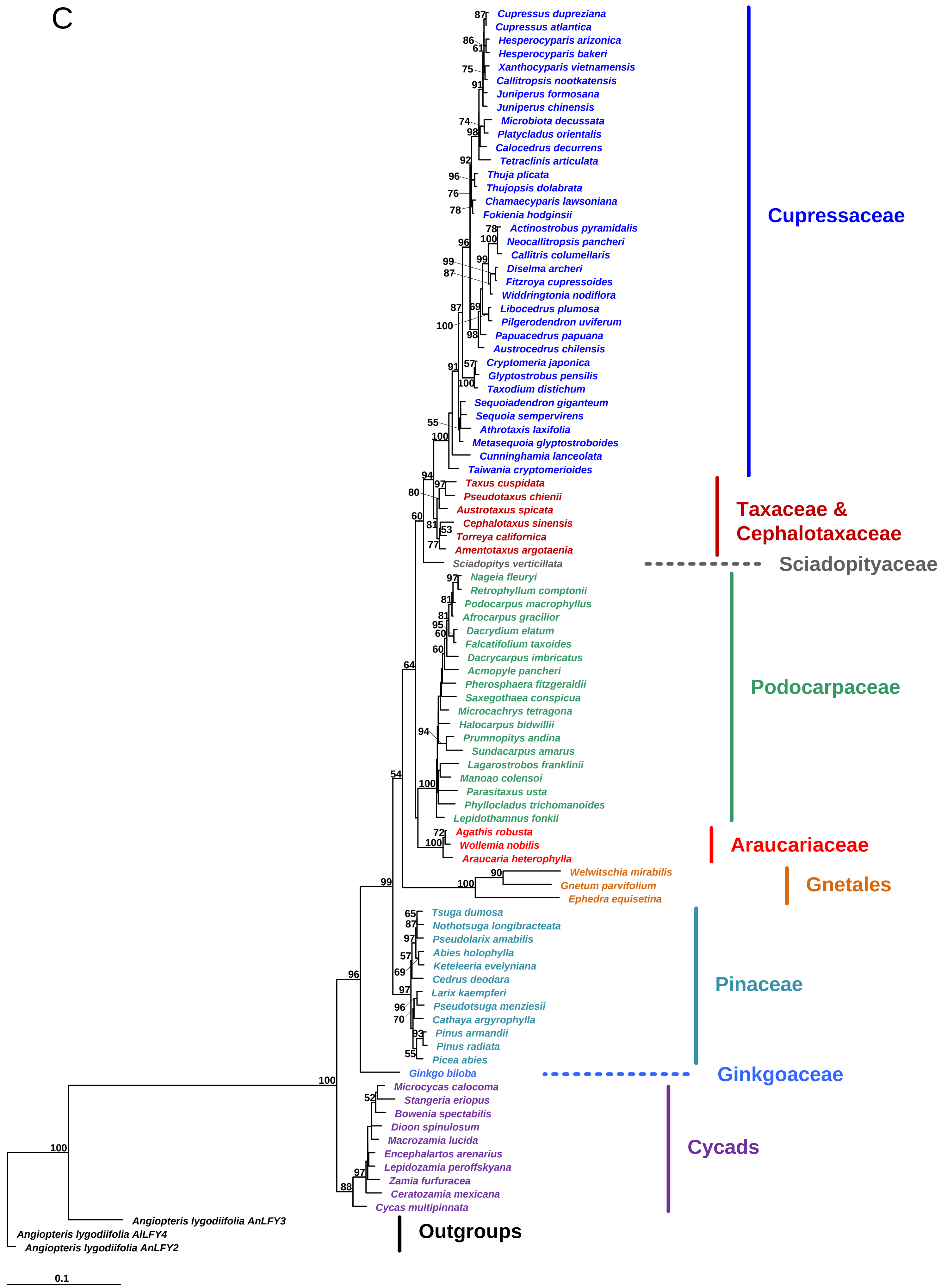
C
Cupressus dupreziana
Cupressus atlantica
Hesperocyparis arizonica
Hesperocyparis bakeri
Xanthocyparis vietnamensis
Callitropsis nootkatensis
Juniperus formosana
Juniperus chinensis
Microbiota decussata
Platycladus orientalis
Calocedrus decurrens
Tetraclinis articulata
Thuja plicata
Thujopsis dolabrata
Chamaecyparis lawsoniana
Fokienia hodginsii
Actinostrobus pyramidalis
Neocallitropsis pancheri
Callitris columellaris
Diselma archeri
Fitzroya cupressoides
Widdringtonia nodiflora
Libocedrus plumosa
Pilgerodendron uviferum
Papuacedrus papuana
Austrocedrus chilensis
Cryptomeria japonica
Glyptostrobus pensilis
Taxodium distichum
Sequoiadendron giganteum
Sequoia sempervirens
Athrotaxis laxifolia
Metasequoia glyptostroboides
Cunninghamia lanceolata
Taiwania cryptomerioides
Taxus cuspidata
Pseudotaxus chienii
Austrotaxus spicata
Cephalotaxus sinensis
Torreya californica
Amentotaxus argotaenia
Sciadopitys verticillata
Nageia fleuryi
Retrophyllum comptonii
Podocarpus macrophyllus
Afrocarpus gracilior
Dacrydium elatum
Falcatifolium taxoides
Dacrycarpus imbricatus
Acmopyle pancheri
Pherosphaera fitzgeraldii
Saxegothaea conspicua
Microcachrys tetragona
Halocarpus bidwillii
Prumnopitys andina
Sundacarpus amarus
Lagarostrobos franklinii
Manoao colensoi
Parasitaxus usta
Phyllocladus trichomanoides
Lepidothamnus fonkii
Agathis robusta
Wollemia nobilis
Araucaria heterophylla
Welwitschia mirabilis
Gnetum parvifolium
Ephedra equisetina
Tsuga dumosa
Nothotsuga longibracteata
Pseudolarix amabilis
Abies holophylla
Keteleeria evelyniana
Cedrus deodara
Larix kaempferi
Pseudotsuga menziesii
Cathaya argyrophylla
Pinus armandii
Pinus radiata
Picea abies
Ginkgo biloba
Microcycas calocoma
Stangeria eriopus
Bowenia spectabilis
Dioon spinulosum
Macrozamia lucida
Encephalartos arenarius
Lepidozamia peroffskyana
Zamia furfuracea
Ceratozamia mexicana
Cycas multipinnata
Angiopteris lygodiifolia AnLFY3
Angiopteris lygodiifolia AlLFY4
Angiopteris lygodiifolia AnLFY2
Cupressaceae
Taxaceae &
Cephalotaxaceae
Sciadopityaceae
Podocarpaceae
Araucariaceae
Gnetales
Pinaceae
Ginkgoaceae
Cycads
Outgroups
0.1

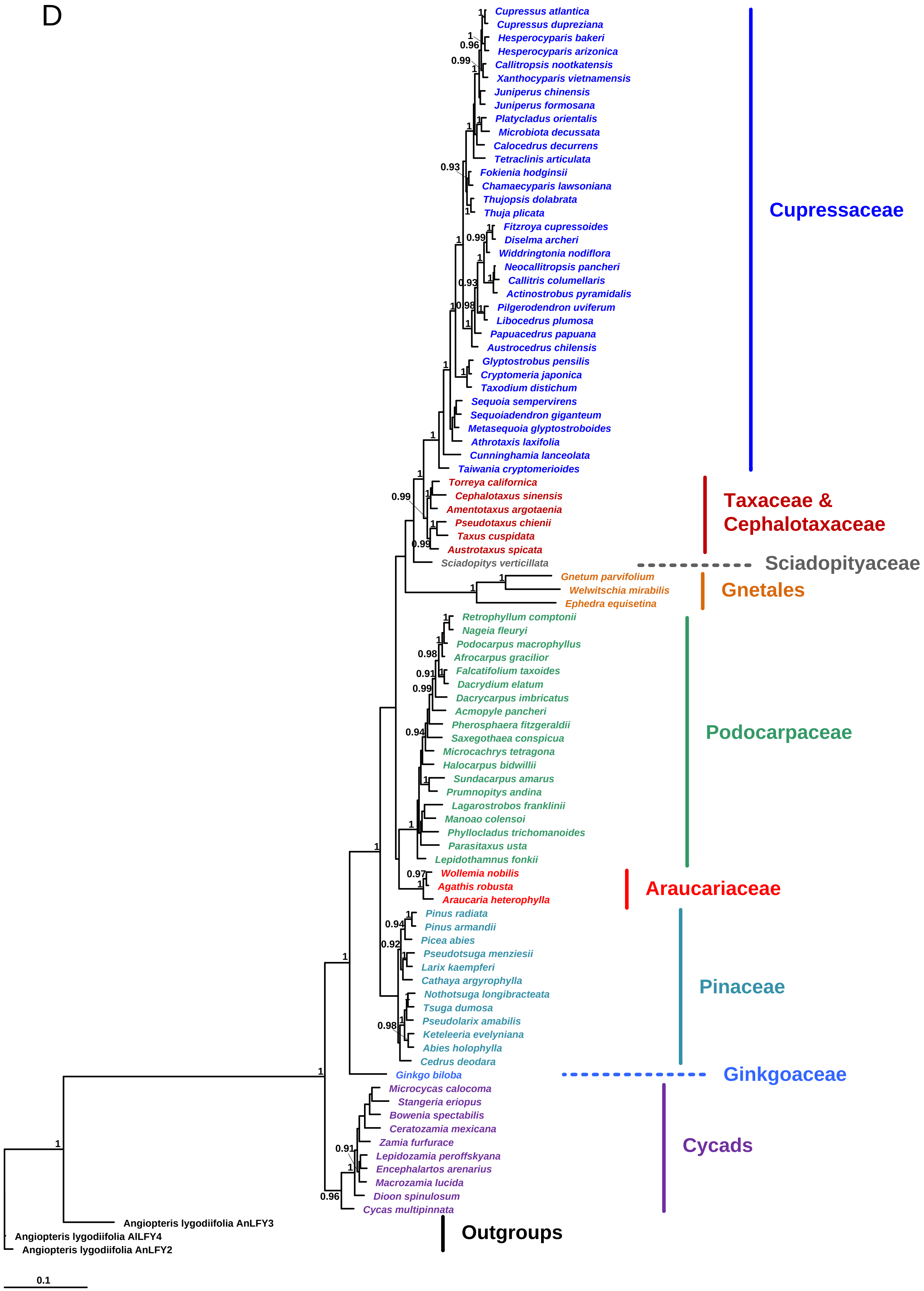
D
Cupressus atlantica
Cupressus dupreziana
Hesperocyparis bakeri
Hesperocyparis arizonica
Callitropsis nootkatensis
Xanthocyparis vietnamensis
Juniperus chinensis
Juniperus formosana
Platycladus orientalis
Microbiota decussata
Calocedrus decurrens
Tetraclinis articulata
Fokienia hodginsii
Chamaecyparis lawsoniana
Thujopsis dolabrata
Thuja plicata
Fitzroya cupressoides
Diselma archeri
Widdringtonia nodiflora
Neocallitropsis pancheri
Callitris columellaris
Actinostrobus pyramidalis
Pilgerodendron uviferum
Libocedrus plumosa
Papuacedrus papuana
Austrocedrus chilensis
Glyptostrobus pensilis
Cryptomeria japonica
Taxodium distichum
Sequoia sempervirens
Sequoiadendron giganteum
Metasequoia glyptostroboides
Athrotaxis laxifolia
Cunninghamia lanceolata
Taiwania cryptomerioides
Torreya californica
Cephalotaxus sinensis
Amentotaxus argotaenia
Pseudotaxus chienii
Taxus cuspidata
Austrotaxus spicata
Sciadopitys verticillata
Gnetum parvifolium
Welwitschia mirabilis
Ephedra equisetina
Retrophyllum comptonii
Nageia fleuryi
Podocarpus macrophyllus
Afrocarpus gracilior
Falcatifolium taxoides
Dacrydium elatum
Dacrycarpus imbricatus
Acmopyle pancheri
Pherosphaera fitzgeraldii
Saxegothaea conspicua
Microcachrys tetragona
Halocarpus bidwillii
Sundacarpus amarus
Prumnopitys andina
Lagarostrobos franklinii
Manoao colensoi
Phyllocladus trichomanoides
Parasitaxus usta
Lepidothamnus fonkii
Wollemia nobilis
Agathis robusta
Araucaria heterophylla
Pinus radiata
Pinus armandii
Picea abies
Pseudotsuga menziesii
Larix kaempferi
Cathaya argyrophylla
Nothotsuga longibracteata
Tsuga dumosa
Pseudolarix amabilis
Keteleeria evelyniana
Abies holophylla
Cedrus deodara
Ginkgo biloba
Microcycas calocoma
Stangeria eriopus
Bowenia spectabilis
Ceratozamia mexicana
Zamia furfurace
Lepidozamia peroffskyana
Encephalartos arenarius
Macrozamia lucida
Dioon spinulosum
Cycas multipinnata
Angiopteris lygodiifolia AnLFY3
Angiopteris lygodiifolia AlLFY4
Angiopteris lygodiifolia AnLFY2
Cupressaceae
Taxaceae &
Cephalotaxaceae
Sciadopityaceae
Gnetales
Podocarpaceae
Araucariaceae
Pinaceae
Ginkgoaceae
Cycads
Outgroups
0.1

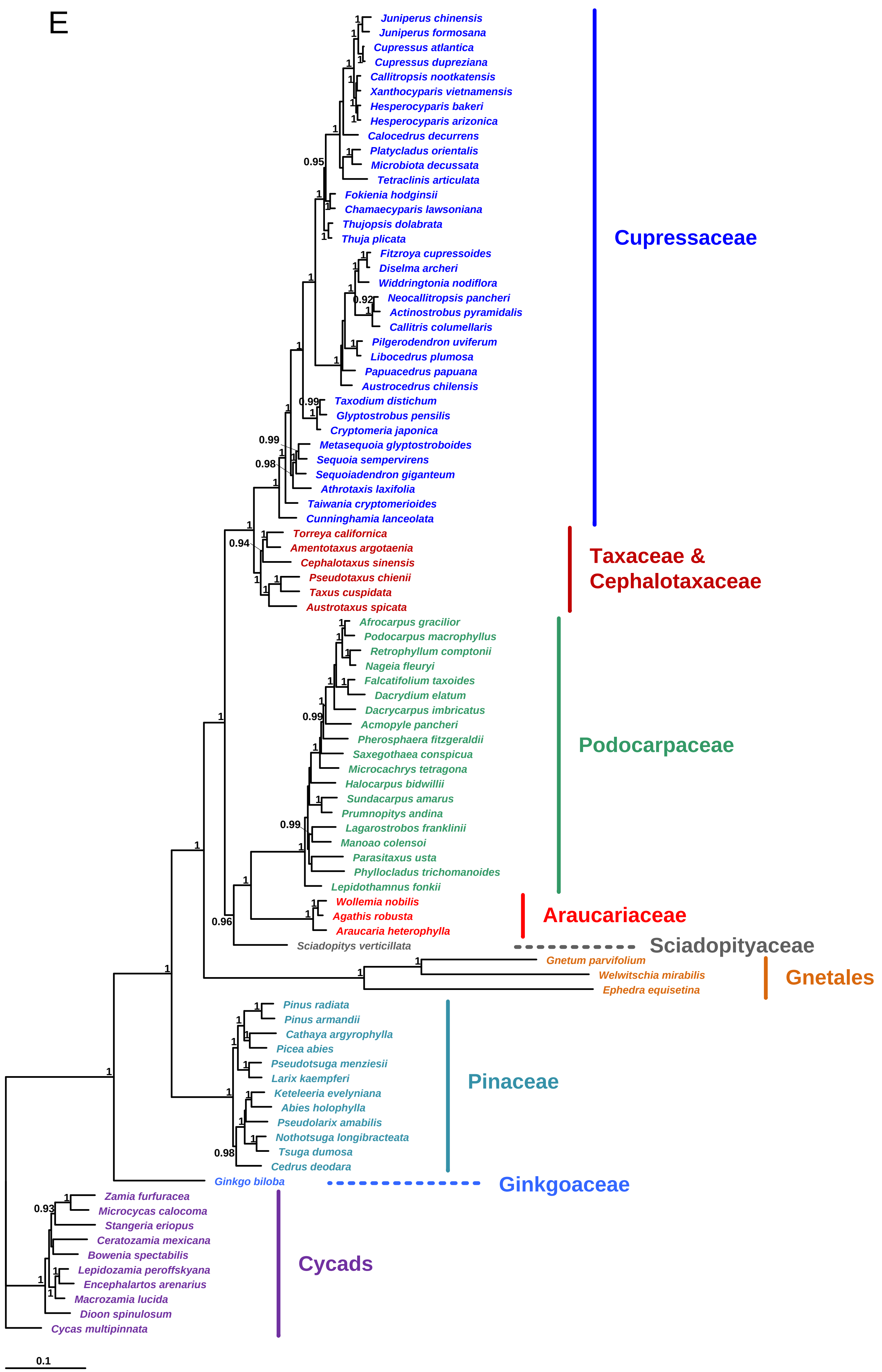

E
Juniperus chinensis
Juniperus formosana
Cupressus atlantica
Cupressus dupreziana
Callitropsis nootkatensis
Xanthocyparis vietnamensis
Hesperocyparis bakeri
Hesperocyparis arizonica
Calocedrus decurrens
Platycladus orientalis
Microbiota decussata
Tetraclinis articulata
Fokienia hodginsii
Chamaecyparis lawsoniana
Thujopsis dolabrata
Thuja plicata
Fitzroya cupressoides
Diselma archeri
Widdringtonia nodiflora
Neocallitropsis pancheri
Actinostrobus pyramidalis
Callitris columellaris
Pilgerodendron uviferum
Libocedrus plumosa
Papuacedrus papuana
Austrocedrus chilensis
Taxodium distichum
Glyptostrobus pensilis
Cryptomeria japonica
Metasequoia glyptostroboides
Sequoia sempervirens
Sequoiadendron giganteum
Athrotaxis laxifolia
Taiwania cryptomerioides
Cunninghamia lanceolata
Cupressaceae
Torreya californica
Amentotaxus argotaenia
Cephalotaxus sinensis
Pseudotaxus chienii
Taxus cuspidata
Austrotaxus spicata
Taxaceae &
Cephalotaxaceae
Afrocarpus gracilior
Podocarpus macrophyllus
Retrophyllum comptonii
Nageia fleuryi
Falcatifolium taxoides
Dacrydium elatum
Dacrycarpus imbricatus
Acmopyle pancheri
Pherosphaera fitzgeraldii
Saxegothaea conspicua
Microcachrys tetragona
Halocarpus bidwillii
Sundacarpus amarus
Prumnopitys andina
Lagarostrobos franklinii
Manoao colensoi
Parasitaxus usta
Phyllocladus trichomanoides
Lepidothamnus fonkii
Podocarpaceae
Wollemia nobilis
Agathis robusta
Araucaria heterophylla
Araucariaceae
Sciadopitys verticillata
Sciadopityaceae
Gnetum parvifolium
Welwitschia mirabilis
Ephedra equisetina
Gnetales
Pinus radiata
Pinus armandii
Cathaya argyrophylla
Picea abies
Pseudotsuga menziesii
Larix kaempferi
Keteleeria evelyniana
Abies holophylla
Pseudolarix amabilis
Nothotsuga longibracteata
Tsuga dumosa
Cedrus deodara
Pinaceae
Ginkgo biloba
Ginkgoaceae
Zamia furfuracea
Microcycas calocoma
Stangeria eriopus
Ceratozamia mexicana
Bowenia spectabilis
Lepidozamia peroffskyana
Encephalartos arenarius
Macrozamia lucida
Dioon spinulosum
Cycas multipinnata
Cycads
0.1

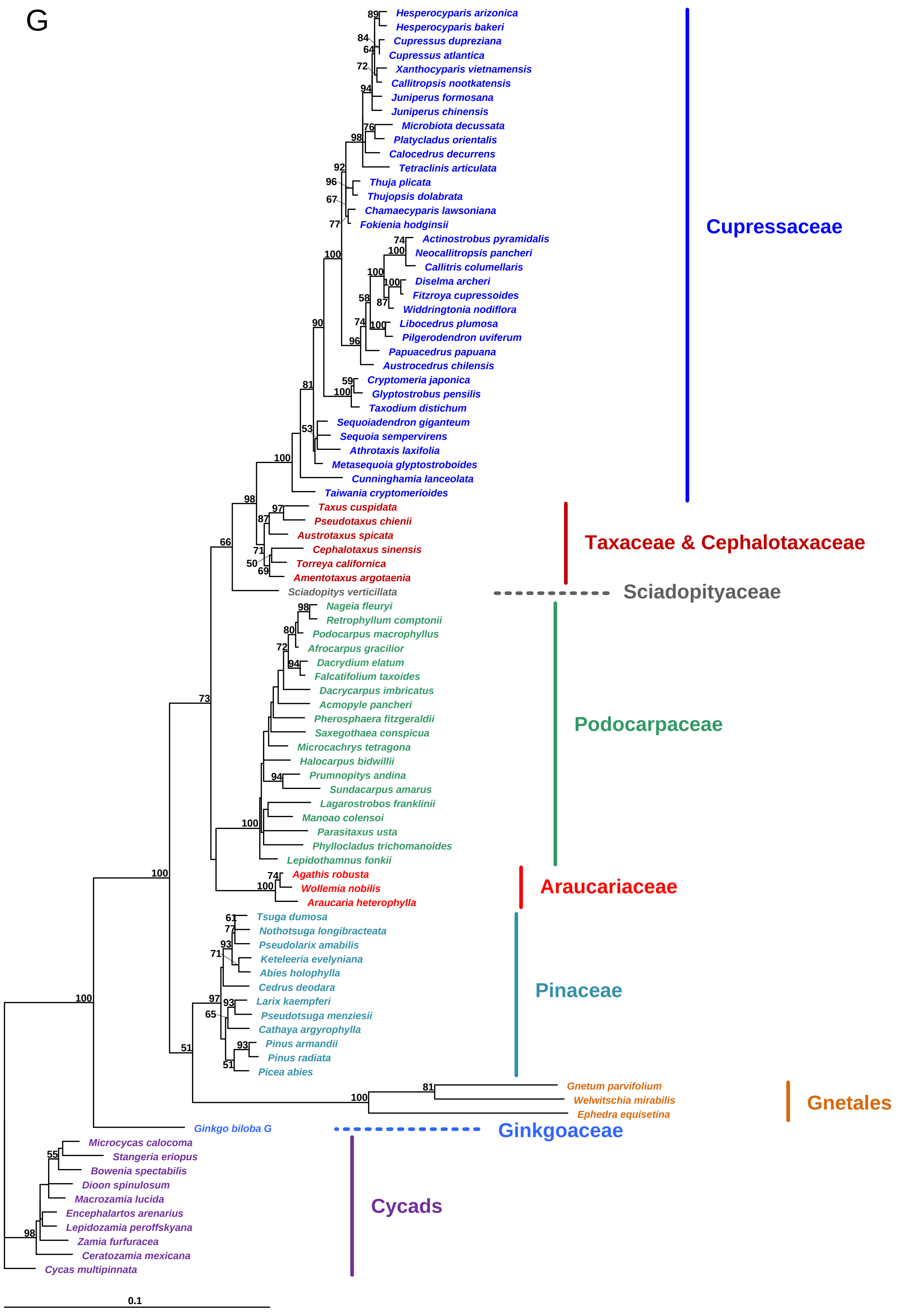
G
Hesperocyparis arizonica
Hesperocyparis bakeri
Cupressus dupreziana
Cupressus atlantica
Xanthocyparis vietnamensis
Callitropsis nootkatensis
Juniperus formosana
Juniperus chinensis
Microbiota decussata
Platycladus orientalis
Calocedrus decurrens
Tetraclinis articulata
Thuja plicata
Thujopsis dolabrata
Chamaecyparis lawsoniana
Fokienia hodginsii
Actinostrobus pyramidalis
Neocallitropsis pancheri
Callitris columellaris
Diselma archeri
Fitzroya cupressoides
Widdringtonia nodiflora
Libocedrus plumosa
Pilgerodendron uviferum
Papuacedrus papuana
Austrocedrus chilensis
Cryptomeria japonica
Glyptostrobus pensilis
Taxodium distichum
Sequoiadendron giganteum
Sequoia sempervirens
Athrotaxis laxifolia
Metasequoia glyptostroboides
Cunninghamia lanceolata
Taiwania cryptomerioides
Taxus cuspidata
Pseudotaxus chienii
Austrotaxus spicata
Cephalotaxus sinensis
Torreya californica
Amentotaxus argotaenia
Sciadopitys verticillata
Nageia fleuryi
Retrophyllum comptonii
Podocarpus macrophyllus
Afrocarpus gracilior
Dacrydium elatum
Falcatifolium taxoides
Dacrycarpus imbricatus
Acmopyle pancheri
Pherosphaera fitzgeraldii
Saxegothaea conspicua
Microcachrys tetragona
Halocarpus bidwillii
Prumnopitys andina
Sundacarpus amarus
Lagarostrobos franklinii
Manoao colensoi
Parasitaxus usta
Phyllocladus trichomanoides
Lepidothamnus fonkii
Agathis robusta
Wollemia nobilis
Araucaria heterophylla
Tsuga dumosa
Nothotsuga longibracteata
Pseudolarix amabilis
Keteleeria evelyniana
Abies holophylla
Cedrus deodara
Larix kaempferi
Pseudotsuga menziesii
Cathaya argyrophylla
Pinus armandii
Pinus radiata
Picea abies
Gnetum parvifolium
Welwitschia mirabilis
Ephedra equisetina
Ginkgo biloba G
Microcycas calocoma
Stangeria eriopus
Bowenia spectabilis
Dioon spinulosum
Macrozamia lucida
Encephalartos arenarius
Lepidozamia peroffskyana
Zamia furfuracea
Ceratozamia mexicana
Cycas multipinnata
Cupressaceae
Taxaceae & Cephalotaxaceae
Sciadopityaceae
Podocarpaceae
Araucariaceae
Pinaceae
Gnetales
Ginkgoaceae
Cycads
0.1

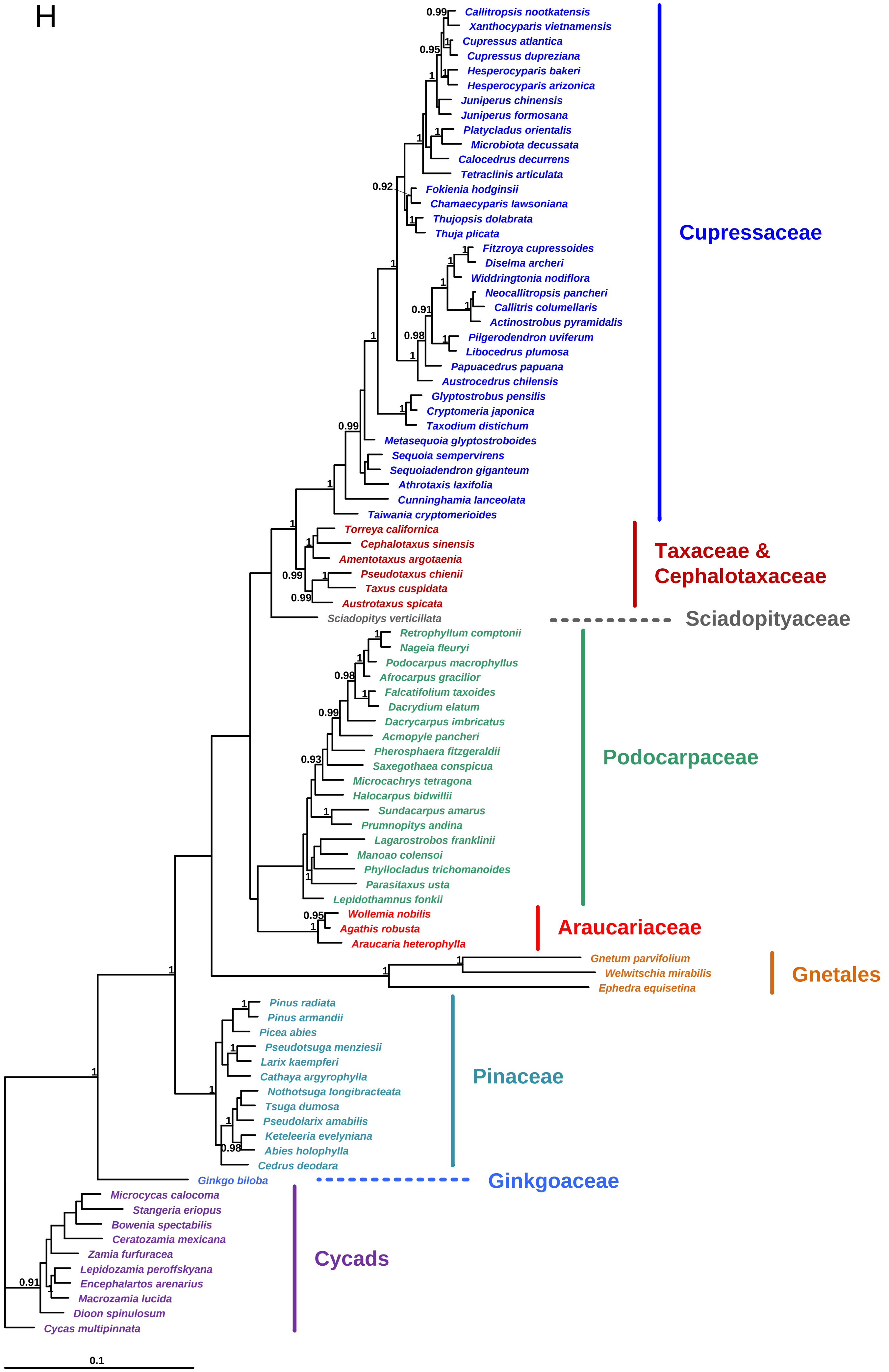
H
Callitropsis nootkatensis
Xanthocyparis vietnamensis
Cupressus atlantica
Cupressus dupreziana
Hesperocyparis bakeri
Hesperocyparis arizonica
Juniperus chinensis
Juniperus formosana
Platycladus orientalis
Microbiota decussata
Calocedrus decurrens
Tetraclinis articulata
Fokienia hodginsii
Chamaecyparis lawsoniana
Thujopsis dolabrata
Thuja plicata
Fitzroya cupressoides
Diselma archeri
Widdringtonia nodiflora
Neocallitropsis pancheri
Callitris columellaris
Actinostrobus pyramidalis
Pilgerodendron uviferum
Libocedrus plumosa
Papuacedrus papuana
Austrocedrus chilensis
Glyptostrobus pensilis
Cryptomeria japonica
Taxodium distichum
Metasequoia glyptostroboides
Sequoia sempervirens
Sequoiadendron giganteum
Athrotaxis laxifolia
Cunninghamia lanceolata
Taiwania cryptomerioides
Cupressaceae
Torreya californica
Cephalotaxus sinensis
Amentotaxus argotaenia
Pseudotaxus chienii
Taxus cuspidata
Austrotaxus spicata
Taxaceae & Cephalotaxaceae
Sciadopitys verticillata
Sciadopityaceae
Retrophyllum comptonii
Nageia fleuryi
Podocarpus macrophyllus
Afrocarpus gracilior
Falcatifolium taxoides
Dacrydium elatum
Dacrycarpus imbricatus
Acmopyle pancheri
Pherosphaera fitzgeraldii
Saxegothaea conspicua
Microcachrys tetragona
Halocarpus bidwillii
Sundacarpus amarus
Prumnopitys andina
Lagarostrobos franklinii
Manoao colensoi
Phyllocladus trichomanoides
Parasitaxus usta
Lepidothamnus fonkii
Podocarpaceae
Wollemia nobilis
Agathis robusta
Araucaria heterophylla
Araucariaceae
Gnetum parvifolium
Welwitschia mirabilis
Ephedra equisetina
Gnetales
Pinus radiata
Pinus armandii
Picea abies
Pseudotsuga menziesii
Larix kaempferi
Cathaya argyrophylla
Nothotsuga longibracteata
Tsuga dumosa
Pseudolarix amabilis
Keteleeria evelyniana
Abies holophylla
Cedrus deodara
Pinaceae
Ginkgo biloba
Ginkgoaceae
Microcycas calocoma
Stangeria eriopus
Bowenia spectabilis
Ceratozamia mexicana
Zamia furfuracea
Lepidozamia peroffskyana
Encephalartos arenarius
Macrozamia lucida
Dioon spinulosum
Cycas multipinnata
Cycads
0.1

Fig. S1. The ML and BI trees of gymnosperms constructed from combined *LFY* and *NLY* sequences. Numbers associated with branches are bootstrap percentages of ML higher than 50% and Bayesian posterior probabilities greater than 0.90, respectively. A, ML tree from the CDS sequences with *Angiopteris lygodiifolia* as outgroup; B, BI tree from the CDS sequences with *Angiopteris lygodiifolia* as outgroup; C, ML tree from the 1st + 2nd codon positions with *Angiopteris lygodiifolia* as outgroup; D, BI tree from the 1st + 2nd codon positions with *Angiopteris lygodiifolia* as outgroup; E, BI tree from the CDS sequences with cycads as functional outgroups; F, ML tree from the 1st + 2nd codon positions with cycads as functional outgroups; G, BI tree from the 1st + 2nd codon positions with cycads as functional outgroups.
