# Supplementary material for: Phylogeny and Divergence Times of Gymnosperms Inferred from Single-Copy Nuclear Genes
Source: PLoS One. 2014 Sep 15;9(9):e107679. doi: 10.1371/journal.pone.0107679 (PMC4164646; doi:10.1371/journal.pone.0107679)
Supplement: Table S1 — Sources of materials. (DOC) [file pone.0107679.s002.doc]

**Table S1.** Sources of materials.

| **Family** | **Species** | **Sources/vouchers or living accessions** | **GenBank Accessions** | |
| --- | --- | --- | --- | --- |
|  |  |  | ***LFY*** | ***NLY*** |
| **Pinaceae** | *Abies holophylla* Maxim. | Yang et al. (2012) | HQ245864 | HQ245773 |
|  | *Cathaya argyrophylla* Chun & Kuang | Dayao Mountains, Guangxi, China/Wang, X.-Q DY08 | KF377885 | KF377949 |
|  | *Cedrus deodara* (Roxb.) *G. Don* | Yang et al. (2012) | HQ245865 | HQ245774 |
|  | *Keteleeria evelyniana* Mast. | Hangzhou Botanical Garden, Zhejiang, China | KF377887 | KF377945 |
|  | *Larix kaempferi* (Lamb.) Carriere | Botanical Garden, Institute of Botany, Beijing, China | KF377890  KF377891 | KF377948 |
|  | *Nothotsuga longibracteata* (W. C. Cheng) Hu ex C. N. Page | Xinning, Hunan, China/LuoZC-002 | KF377888 | KF377947 |
|  | *Picea abies* (L.) H. Karst. | Yang et al. (2012) | AY701763 | AY701762 |
|  | *Pinus armandii* Franch. | Yang et al. (2012) | HQ245873 | HQ245782 |
|  | *Pinus radiata* D. Don | Yang et al. (2012) | U92008.1 | U76757.1 |
|  | *Pseudolarix amabilis* (J. Nelson) Rehder | Zheshan Park, Wuhu, Anhui, China | KF377889 | KF377944 |
|  | *Pseudotsuga menziesii* (Mirb.) Franco | Botanical Garden, Institute of Botany, Beijing, China | KF377892 | KF377950 |
|  | *Tsuga dumosa* (D.Don) Eichler | Yaojiaping, Sichuan, China/YJP30 | KF377886 | KF377946 |
| **Araucariaceae** | *Agathis robusta* (C. Moore ex F. Muell.) F. Muell. | Royal Botanical Garden, Melbourne, Australia/ 2011072712-1 | KF377856 | KF377913 |
|  | *Araucaria heterophylla* (Salisb.) Franco | Yang et al. (2012) | HQ245859 | HQ245769 |
|  | *Wollemia nobilis* W. G. Jones et al. | Royal Botanical Garden, Melbourne, Australia/20071012 | KF377857 | KF377914 |
| **Cephalotaxaceae** | *Cephalotaxus sinensis* (Rehder & E. H. Wilson) H. L. Li | Yang et al. (2012) | HQ245860 | HQ245770 |
| **Cupressaceae *s.l.*** | *Actinostrobus pyramidalis* Miq. | Yang et al. (2012) | HQ245806 | HQ245712 |
|  | *Athrotaxis laxifolia* Hook. | Yang et al. (2012) | HQ245807 | HQ245713 |
|  | *Austrocedrus chilensis* (D. Don) Pic. Serm. & Bizzarri | Yang et al. (2012) | HQ245808 | HQ245714 |
|  | *Callitris columellaris* F. Muell. | Yang et al. (2012) | HQ245809 | HQ245715 |
|  | *Callitropsis nootkatensis* (D. Don) Oersted ex D. P. Little | Yang et al. (2012) | HQ245814 | HQ245720 |
|  | *Calocedrus decurrens* (Torr.) Florin | Yang et al. (2012) | HQ245812 | HQ245718 |
|  | *Chamaecyparis lawsoniana* (A. Murray bis) Parl. | Yang et al. (2012) | HQ245815 | HQ245722 |
|  | *Cryptomeria japonica* (Thunb. ex L. f.) D. Don | Yang et al. (2012) | HQ245816 | HQ245723 |
|  | *Cunninghamia lanceolata* (Lamb.) Hook. | Yang et al. (2012) | HQ245817 | HQ245724 |
|  | *Cupressus atlantica* Gaussen | Yang et al. (2012) | HQ245819 | HQ245725 |
|  | *Cupressus dupreziana* A. Camus | Yang et al. (2012) | HQ245821 | HQ245726 |
|  | *Diselma archeri* Hook. f. | Yang et al. (2012) | HQ245823 | HQ245730 |
|  | *Fitzroya cupressoides* (Molina) I. M. Johnst. | Yang et al. (2012) | HQ245824  HQ245825 | HQ245731  HQ245732 |
|  | *Fokienia hodginsii* (Dunn) A. Henry & H. H. Thomas | Yang et al. (2012) | HQ245826 | HQ245733 |
|  | *Glyptostrobus pensilis* (Staunton ex D. Don) K. Koch | Yang et al. (2012) | HQ245827 | HQ245734 |
|  | *Hesperocyparis arizonica* (Greene) Bartel | Yang et al. (2012) | HQ245828 | HQ245735 |
|  | *Hesperocyparis bakeri* (Jeps.) Bartel | Yang et al. (2012) | HQ245829  HQ245830 | HQ245736  HQ245737 |
|  | *Juniperus chinensis* L. | Yang et al. (2012) | HQ245833 | HQ245740 |
|  | *Juniperus formosana* Hayata | Yang et al. (2012) | HQ245834 | HQ245742 |
|  | *Libocedrus plumosa* (D. Don) Sarg. | Yang et al. (2012) | HQ245839 | HQ245747 |
|  | *Metasequoia glyptostroboides* Hu & W. C. Cheng | Yang et al. (2012) | HQ245841 | HQ245749 |
|  | *Microbiota decussata* Kom. | Yang et al. (2012) | HQ245842 | HQ245750 |
|  | *Neocallitropsis pancheri* (Carrière) de Laub. | Yang et al. (2012) | HQ245843 | HQ245751 |
|  | *Papuacedrus papuana* (F. Muell.) H. L. Li | Yang et al. (2012) | HQ245844 | HQ245752 |
|  | *Pilgerodendron uviferum* (D. Don) Florin | Yang et al. (2012) | HQ245845 | HQ245753 |
|  | *Platycladus orientalis* (L.) Franco | Yang et al. (2012) | HQ245846 | HQ245754 |
|  | *Sequoia sempervirens* (D. Don) Endl. | Yang et al. (2012) | HQ245847  HQ245848 | HQ245755  HQ245756 |
|  | *Sequoiadendron giganteum* (Lindl.) J. Buchholz | Yang et al. (2012) | HQ245849 | HQ245757 |
|  | *Taiwania cryptomerioides* Hayata | Yang et al. (2012) | HQ245850 | HQ245758 |
|  | *Taxodium distichum* var. *imbricatu* (Nutt.) Croom | Yang et al. (2012) | HQ245851 | HQ245759 |
|  | *Tetraclinis articulata* (Vahl) Mast. | Yang et al. (2012) | HQ245852 | HQ245760 |
|  | *Thuja plicata* Donn ex D. Don | Yang et al. (2012) | HQ245854 | HQ245763 |
|  | *Thujopsis dolabrata* (Thunb. ex L. f.) Siebold & Zucc. | Yang et al. (2012) | HQ245855 | HQ245764 |
|  | *Widdringtonia nodiflora* (L.) Powrie | Yang et al. (2012) | HQ245856 | HQ245765 |
|  | *Xanthocyparis vietnamensis* Farjón & H. T. Nguyễn | Yang et al. (2012) | HQ245857  HQ245858 | HQ245767  HQ245768 |
| **Podocarpaceae** | *Acmopyle pancheri* (Brongn. & Gris) Pilg. | Royal Botanic Garden, Edinburgh/19842681 A | KF377879 | KF377929  KF377930 |
|  | *Afrocarpus gracilior* (Pilg.) C. N. Page | National Botanic Garden of Belgium, Dutch/ ET-0-BR-19700726 | KF377875 | KF377926  KF377927 |
|  | *Dacrycarpus imbricatus* (Blume) de Laub. | Xishuangbanna Tropical Botanical Garden, Yunnan, China/20111029 | KF377865 | KF377921  KF377922 |
|  | *Dacrydium elatum* (Roxb.) Wall. ex Hook. | Hainan, China / WZS04 | KF377866  KF377867 | KF377925 |
|  | *Falcatifolium taxoides* (Brongn. & Gris) de Laub. | Royal Botanic Garden, Edinburgh/19842581 A | KF377869 | KF377924 |
|  | *Halocarpus bidwillii* (Hook. f. ex. Kirk) Quinn | Royal Botanic Garden, Edinburgh/19832579 A | KF377883  KF377884 | KF377942 |
|  | *Lagarostrobos franklinii* (Hook. f.) Quinn | Royal Botanic Garden, Edinburgh/19763971 A17 | KF377881 | KF377937 |
|  | *Lepidothamnus fonkii* Phil. | Royal Botanic Garden, Edinburgh/20060725 CT | KF377872 | KF377932  KF377933 |
|  | *Manoao colensoi* (Hook.) Molly | Royal Botanic Garden, Edinburgh/19842513 A | KF377868 | KF377941 |
|  | *Microcachrys tetragona* (Hook.) Hook. f. | Royal Botanic Garden, Edinburgh/19771626 A | KF377880 | KF377935  KF377936 |
|  | [*Nageia fleuryi* (Hickel) de Laub.](http://www.discoverlife.org/mp/20q?search=Nageia+fleuryi) | Hangzhou Botanical Garden, Hangzhou, China/ Ran07029 | KF377863  KF377864 | KF377923 |
|  | *Parasitaxus usta* (Vieill.) de Laub. | Royal Botanic Garden, Kew/37649 | KF377874 | KF377912 |
|  | *Pherosphaera fitzgeraldii* (F. Muell.) Hook. f. | Royal Botanic Garden, Kew/804 | KF377873 | KF377931 |
|  | *Phyllocaldus trichomanoides* D. Don var. trichomanoides | Royal Botanic Garden, Kew/24194 | KF377877  KF377878 | KF377943 |
|  | *Podocarpus macrophyllus* (Thunb.) Sweet | Botanical Garden, Institute of Botany, Beijing, China/ Yang06013 | HQ245862 | submitted |
|  | *Prumnopitys andina* (Poepp. ex Endl.) de Laub. | Royal Botanic Garden, Edinburgh/20071036 | KF377871 | KF377940 |
|  | *Retrophyllum comptonii* (J. Buchholz) C. N. Page | Royal Botanic Garden, Edinburgh/20060947 H | KF377870 | KF377928 |
|  | *Saxegothaea conspicua* Lindl. | National Botanic Garden of Belgium, Dutch/ CL-0-BR-19850152 | KF377876 | KF377934 |
|  | *Sundacarpus amarus* (Blume) C. N. Page | Montgomery Botanical Center, USA/20060819*A | KF377882 | KF377938  KF377939 |
| **Sciadopityaceae** | *Sciadopitys verticillata* (Thunb.) Siebold & Zucc. | Yang et al. (2012) | HQ245863 | HQ245772 |
| **Taxaceae** | *Amentotaxus argotaenia* (Hance) Pilg. | Xingshan, Hubei, China/11282a | KF377859 | KF377915 |
|  | *Austrotaxus spicata* R. H. Compton | New Caledonia/CAGNC69 2009 9 14 | KF377860 | KF377917 |
|  | *Pseudotaxus chienii* (W. C. Cheng) W. C. Cheng | Lushan Botanical Garden, Jiangxi, China/Ran07023 | KF377858 | KF377918 |
|  | *Taxus cuspidata* var. *nana* Hort. ex Rehder | Yang et al. (2012) | HQ245861 | HQ245771 |
|  | *Torreya californica* Torr. | Jordan Botanic Garden, Geneva, Switzerland/WXQ2142 | KF377861  KF377862 | KF377916 |
| **Ephedraceae** | *Ephedra equisetina* Bunge | Botanical Garden, Institute of Botany, Beijing | KF377910  KF377911 | KF377908  KF377909 |
| **Gnetaceae** | *Gnetum parvifolium* (Warb.) C. Y. Cheng ex Chun | Shindo et al. (Unpublished) | AB022667 |  |
| **Welwitschiaceae** | *Welwitschia mirabilis* Hook. f. | Frohlich and Parker (2000) | AF109130 | AF072369 |
| **Ginkgoaceae** | *Ginkgo biloba* L. | Frohlich and Parker (2000) | AF108228 | AF105111 |
| **Cycadaceae** | *Cycas multipinnata* C. J. Chen & S. Y. Yang | Xishuangbanna Tropical Botanical Garden, Yunnan, China | KF377901 | KF377963 |
| **Zamiaceae** | *Bowenia spectabilis* Hook. ex Hook. f. | Shenzhen Fairy Lake Botanical Garden, Shenzhen, China | KF377904  KF377905 | KF377962 |
|  | *Ceratozamia mexicana* Brongn. | Royal Botanical Garden, Melbourne, Australia/ 2011072701 | KF377897  KF377898 | KF377956 |
|  | *Dioon spinulosum* Dyer ex Eichl. | Xishuangbanna Tropical Botanical Garden, Yunnan, China | KF377899  KF377900 | KF377957  KF377958 |
|  | *Encephalartos arenarius* R. A. Dyer | Royal Botanical Garden Melbourne, Australia/ 2011072708 | KF377893  KF377894 | KF377951  KF377952 |
|  | *Lepidozamia peroffskyana* Regel | Royal Botanical Garden Melbourne, Australia/ 2011072702-1 | KF377895 | KF377953 |
|  | *Macrozamia moorei* F. Muell. | Xishuangbanna Tropical Botanical Garden, Yunnan, China | KF377896 | KF377954  KF377955 |
|  | *Microcycas calocoma* (Miq.) A. DC. | Shenzhen Fairy Lake Botanical Garden, Shenzhen, China | KF377906 | KF377961 |
|  | *Stangeria eriopus* (Kunze) Baill. | Shenzhen Fairy Lake Botanical Garden, Shenzhen, China | KF377907 | KF377959  KF377960 |
|  | *Zamia furfuracea* L. f. | Frohlich and Parker (2000) | AF105107 | AF105108 |
| **Outgroups** | *Angiopteris lygodiifolia* | Himi et al. (2001) | AB050091  AB050092  AB085699 |  |

**References**

Frohlich MW, Parker DS. (2000) The mostly male theory of flower evolutionary origins: from genes to fossils. Syst Bot 25: 155-171.

Himi S, Sano R, Nishiyama T, Tanahashi T, Kato M, et al. (2001) Evolution of MADS-box gene induction by *FLO/LFY* genes. J Mol Evol 53: 387-393.

Yang Z-Y, Ran J-H, Wang X-Q. (2012) Three genome-based phylogeny of Cupressaceae *s.l.*: Further evidence for the evolution of gymnosperms and the Southern Hemisphere biogeography. Mol Phylogenet Evol 64: 452-470.
