# Supplementary material for: Phylogeny and Divergence Times of Gymnosperms Inferred from Single-Copy Nuclear Genes
Source: PLoS One. 2014 Sep 15;9(9):e107679. doi: 10.1371/journal.pone.0107679 (PMC4164646; doi:10.1371/journal.pone.0107679)
Supplement: Table S2 — Primers used for the PCR amplification and sequencing. (DOC) [file pone.0107679.s003.doc]

**Table S2.** Primers used for the PCR amplification and sequencing.

| **Primer** | **Sequence (5’-3’)** | **References** |
| --- | --- | --- |
| LFYE1F3 | TGCAGCTTTCTTCAAGTGGGA | Peng and Wang, (2008) |
| LFYE3R3 | TGTGGGAACATACCAAATTCG | Yang et al. (2012) |
| LFYE3R4 | CCAGATTCGAAGCTTTTCATG | Peng and Wang (2008) |
| LFYE3R4’ | TATTCGAAGCTTCTCGTGCTT | this study |
| LFYE3R5 | CCAGCCATTRTCTTTGGCCAT | this study |
| Eph_LF1 | GAACAGGAGGCTTTGCTAAGT | this study |
| Eph_LR1 | GCAGAGGGACAAGATAGAGT | this study |
| NLYE1F1 | AAGGAATTGAAGTCCCTTGAAG | Yang et al. (2012) |
| NLYE1F2 | AGAGTTGTTCAGAGAGTATGGAG | Yang et al. (2012) |
| NLYE1F3 | TACTTGACMATGGCCAAGATGAC | this study |
| NLYE3R3 | CTTTGCTTCTCTCCATATGACAC | Yang et al. (2012) |
| NLYE3R4 | TTGTGGGRACATACCAAATCT | this study |
| CycNE1F1 | AGTTGTTGAAGTCGCTGGAAG | this study |
| CycNE1F2 | TGATGAAGACTTTGGTGGAG | this study |
| CycNE3R1 | CGAAGTTTCTCATTGCGATTG | this study |
| PinNE1F3 | GCCAACACCCTTGTCAATATG | this study |
| Eph_NR4 | GYTGGCGTAGYTTGGTGGGAA | this study |

Peng D, Wang XQ (2008) Reticulate evolution in *Thuja* inferred from multiple gene sequences: implications for the study of biogeographical disjunction between eastern Asia and North America. Mol Phylogenet Evol 47: 1190-1202.

Yang Z-Y, Ran J-H, Wang X-Q (2012) Three genome-based phylogeny of Cupressaceae *s.l.*: Further evidence for the evolution of gymnosperms and Southern Hemisphere biogeography. Mol Phylogenet Evol 64: 452-470.
