# Supplementary material for: Phylogeny and Divergence Times of Gymnosperms Inferred from Single-Copy Nuclear Genes
Source: PLoS One. 2014 Sep 15;9(9):e107679. doi: 10.1371/journal.pone.0107679 (PMC4164646; doi:10.1371/journal.pone.0107679)
Supplement: Table S3 — The eleven calibration points used in divergence time estimation for gymnosperms. All constraints were given lognormal prior distributions, where the minimum age was set by the age of the fossil constraint and 95% confidence interval of the probability distribution extending 20 or 40 million years earlier than the minimum age. (DOC) [file pone.0107679.s004.doc]

**Table S3.** The eleven calibration points used in divergence time estimation for gymnosperms. All constraints were given lognormal prior distributions, where the minimum age was set by the age of the fossil constraint and 95% confidence interval of the probability distribution extending 20 or 40 million years earlier than the minimum age. The calibration points A-K correspond to nodes A-K in Fig. 5.

| **Calibration point** | **Assignment** | **Stratum and age priors(Ma)** | **Setting** | **Fossils** | **Reference** |
| --- | --- | --- | --- | --- | --- |
| A | Crown of Arcogymnospermae | [306.2, 366.8] | Log(mean): 3.5  Log(Stdev): 0.5  Offset: 291.7 | *Cordaixylon iowensis* in the Laddsdale Coals (Cherokee Group, Desmoinesian Series; 307.2 ± 1.0 Ma): near What Cheer, Iowa. *Elkinsia polymorpha* from the Hampshire Formation, West Virginia, VCo Biozone (363.6–366.8 Ma) | Ravn et al. (1984)  Rothwell et al. (1989) Peppers (1996) |
| B | Stem of *Lepidozamia* | ≥33.9 | Log(mean): 2.391  Log(Stdev): 0.5  Offset: 29.1 | *Lepidozamia* from the Eocene of Australia | Hill (1980) |
| C | *Picea-Cathaya* divergence | ≥133 | Log(mean): 3.08  Log(Stdev): 0.5  Offset: 123.4 | *Picea burtonii* from the Apple Bay locality, Vancouver Island, British Columbia , dated to the Valanginian Stage of the Early Cretaceous (~140-133 Ma) | Klymiuk and Stockey (2012) |
| D | *Welwitschia* (*Ephedra* - *Gnetum*) divergence | ≥125 | Log(mean): 3.08  Log(Stdev): 0.5  Offset: 115.4 | *Eoantha zherikhinii* from the Lower Cretaceous (Neocomian). | Krassilov (1982,1986) |
| E | Araucariaceae-Podocarpaceae divergence | ≥172 | Log(mean): 3.08  Log(Stdev): 0.5  Offset: 162.4 | *Brachyphyllum mammilare* from the Aalenian (172-176 Ma) | Harris (1979) |
| F | *Podocarpus-Retrophyllum* divergence | ≥28 | Log(mean): 2.391  Log(Stdev): 0.5  Offset: 23.2 | *Retrophyllum australe* from the West Dale Flora of southwestern Australia, dated to the Middle Eocene to Oligocene. | Hill and Merrifield (1993) |
| G | Taxaceae-Cupressaceae divergence | ≥197 | Log(mean): 3.08  Log(Stdev): 0.5  Offset: 187.4 | *Palaeotaxus rediviva* in the Upper coal bed of the Skromberga Colliery in Scania, Sweden, dated to the Lowermost Jurassic (Hettangian, 201-197 Ma). | Florin (1958) |
| H | *Sequoia* (*Metasequia* - *Sequoiadendron*) divergence | ≥140 | Log(mean): 3.08  Log(Stdev): 0.5  Offset: 130.4 | *Sequoia* from the Magothy, dated to the early Cretaceous | Penny (1947) |
| I | *Glyptostrobus* - *Taxodium* divergence | ≥99 | Log(mean): 3.08  Log(Stdev): 0.5  Offset: 89.4 | *Glyptostrobus* in the Horseshoe Canyon Formation near Drumheller, Alberta, Canada (upper Cretaceous) | Miller (1977) |
| J | *Widdringtonia* (*Diselma* - *Fitzroya*) divergence | ≥95 | Log(mean): 3.08  Log(Stdev): 0.5  Offset: 85.4 | *Widdringtonia* from the Tuscaloosa Formation of Alabama, ca. 95 Ma | McIver (2001) |
| K | *Cupressus-Juniperus* divergence | ≥33.9 | Log(mean): 2.391  Log(Stdev): 0.5  Offset: 29.1 | *Juniperus pauli* from the Ústí Formation, Czech Republic, dated to the Eocene/Oligocene boundary (~33 Ma). | Kvaček (2002) |

**References**

Aulenback KR, LePage BA. (1998) *Taxodium wallisii* sp. nov.: first occurrence of *Taxodium* from the Upper Cretaceous. Int J Plant Sci 159: 367-390.

Florin R. (1958) On Jurassic taxads and conifers from north-western Europe and Eastern Greenland. Acta Hort Berg 17: 257-402.

Harris TM. (1979) The Yorkshire Jurassic Flora. V. Coniferales. British Museum of Natural History, London.

Hill RS. (1980) Three new Eocene cycads from eastern Australia. Aust J Bot 28: 105.

Hill RS, Merrifield HE. (1993) An early Tertiary macroflora from West Dale, southwestern Australia. Alcheringa 17: 285-326.

Klymiuk AA, Stockey RA. (2012) A Lower Cretaceous (Valanginian) seed cone provides the earliest fossil record for *Picea* (Pinaceae). Am J Bot 99: 1069-1082.

Krassilov V A. (1982) Early Cretaceous flora of Mongolia. Palaeontogr B 181: 1-43.

Krassilov V A. (1986) New floral structure from the Lower Cretaceous of Lake Baikal area. Rev Palaeobot Palynol 47: 9-16.

Kvaček Z. (2002) A new juniper from the Palaeogene of central Europe. Feddes Repert 113: 492-502.

McIver EE. (2001) Cretaceous *Widdringtonia* Endl. (Cupressaceae) from North America. Int J Plant Sci 162: 937-961.

Miller CN. (1977) Mesozoic conifers. Bot Rev 43: 217-280.

Penny JS. (1947) Studies on the conifers of the magothy flora. Am J Bot 34: 281-296.

Peppers RA. (1996) Palynological correlation of major Pennsylvanian (Middle and Upper Carboniferous) chronostratigraphic boundaries in the Illinois and other coal basins. Geol Soc Am Mem 188: 1-111.

Ravn RL*,* Swade JW, Howes MR, Gregory JL, Anderson RR. et al. (1984) Stratigraphy of the Cherokee Group and revision of Pennsylvanian stratigraphic nomenclature in Iowa. Iowa Geol Surv TechInf Ser 12: 1-76.

Rothwell GW, Scheckler SE, Gillespie WH. 1989. *Elkinsia* gen. nov., a late Devonian gymnosperm with cupulate ovules. Bot Gaz 150: 170-189.
