# Supplementary material for: Phylogeny and Divergence Times of Gymnosperms Inferred from Single-Copy Nuclear Genes
Source: PLoS One. 2014 Sep 15;9(9):e107679. doi: 10.1371/journal.pone.0107679 (PMC4164646; doi:10.1371/journal.pone.0107679)
Supplement: Table S4 — Results of the Shimodaira-Hasegawa (SH) test and the Kishino-Hasegawa (KH) test. (DOC) [file pone.0107679.s005.doc]

**Table S4.** Results of the Shimodaira-Hasegawa (SH) test and the Kishino-Hasegawa (KH) test.

| **Dataset** | **-ln L** | **Diff -ln L** | **SH-test p** | **KH-test p** |
| --- | --- | --- | --- | --- |
| Gymnosperms (1) |  |  |  |  |
| CDS |  |  |  |  |
| Ginkgoaceae+conifers+Gnetales | 29714.890 | Best |  |  |
| Ginkgoaceae+Cycadales | 29726.911 | 12.021 | 0.044* | 0.078 |
| Sciadopityaceae+Podocarpaceae+Araucariaceae | 29714.890 | Best |  |  |
| Sciadopityaceae+Cupressaceae+Taxaceae | 29717.388 | 2.498 | 0.370 | 0.740 |
| Gnetales+Conifer II | 29714.890 | Best |  |  |
| Gnetales+Pinaceae | 29733.403 | 18.513 | 0.173 | 0.110 |
| Gnetales+conifers | 29731.300 | 16.410 | 0.184 | 0.017* |
| Gnetales+other gymnosperms | 29751.738 | 36.848 | 0.025* | 0.009* |
| CDS (1st+2nd) |  |  |  |  |
| Ginkgoaceae+conifers+Gnetales | 10316.439 | Best |  |  |
| Ginkgoaceae+Cycadales | 10326.699 | 10.260 | 0.209 | 0.418 |
| Sciadopityaceae+Podocarpaceae+Araucariaceae | 10321.048 | 4.610 | 0.378 | 0.733 |
| Sciadopityaceae+Cupressaceae+Taxaceae | 10316.439 | Best |  |  |
| Gnetales+Conifer II | 10316.439 | 4.526 | 0.504 | 0.738 |
| Gnetales+Pinaceae | 10311.913 | Best |  |  |
| Gnetales+conifers | 10320.259 | 8.346 | 0.451 | 0.350 |
| Gnetales+other gymnosperms | 10338.311 | 26.398 | 0.063 | 0.005* |
| Gymnosperms (2) |  |  |  |  |
| CDS |  |  |  |  |
| Sciadopityaceae+Podocarpaceae+Araucariaceae | 27279.407 | Best |  |  |
| Sciadopityaceae+Cupressaceae+Taxaceae | 27281.799 | 2.391 | 0.355 | 0.727 |
| Gnetales+Conifer II | 27279.407 | Best |  |  |
| Gnetales+Pinaceae | 27295.126 | 15.719 | 0.147 | 0.120 |
| Gnetales+conifers | 27304.677 | 25.270 | 0.068 | 0.109 |
| CDS (1st+2nd) |  |  |  |  |
| Sciadopityaceae+Podocarpaceae+Araucariaceae | 9149.990 | 8.773 | 0.256 | 0.523 |
| Sciadopityaceae+Cupressaceae+Taxaceae | 9141.216 | Best |  |  |
| Gnetales+Conifer II | 9140.817 | Best |  |  |
| Gnetales+Pinaceae | 9141.216 | 0.399 | 0.560 | 0.974 |
| Gnetales+conifers | 9146.869 | 6.052 | 0.357 | 0.237 |

Gymnosperms (1): *Angiopteris* as outgroup; Gymnosperms (2): Cycads as functional outgroups; CDS: coding sequence;

1st+2nd: the first and second codon positions; * p<0.05
