# Supplementary material for: Phylogeny and Divergence Times of Gymnosperms Inferred from Single-Copy Nuclear Genes
Source: PLoS One. 2014 Sep 15;9(9):e107679. doi: 10.1371/journal.pone.0107679 (PMC4164646; doi:10.1371/journal.pone.0107679)
Supplement: Table S5 — Ages of selected clades. (DOC) [file pone.0107679.s006.doc]

**Table S5.** Ages of selected clades.

| **Node*** | **Mean (Ma)** | **95%HPD (Ma)** |
| --- | --- | --- |
| A | 353.9 | 307.8-427.0 |
| B | 39.3 | 31.8-50.2 |
| C | 139.9 | 128.4-154.0 |
| D | 146.1 | 122.3-186.9 |
| E | 186.3 | 168.5-213.1 |
| F | 34.8 | 26.9-45.8 |
| G | 211.5 | 198.0-255.1 |
| H | 146 | 135.7-159.7 |
| I | 104.5 | 94.4-118.5 |
| J | 96.7 | 89.9-106.9 |
| K | 39.3 | 32.2-49.3 |
| 1 | 158.1 | 105.8-220.3 |
| 2 | 107.6 | 71.5-151.7 |
| 3 | 342.9 | 306.4-425.3 |
| 4 | 198.4 | 159.4-251.4 |
| 5 | 236.4 | 192.8-292.4 |
| 6 | 51.2 | 18.4-102.3 |
| 7 | 132.7 | 98.2-165.1 |
| 8 | 276.8 | 230.2-336.0 |
| 9 | 144.38 | 83.2-208.4 |
| 10 | 197.6 | 172.9-227.8 |

*, Node numbers correspond to those in Fig. 5
